# Silymarin-Based Therapies in Chronic Liver Disease: A Systematic Review of Biochemical, Cardiometabolic, Steatosis, and Fibrosis-Related Outcomes

**DOI:** 10.3390/nu18213685

**Published:** 2026-11-06

**Authors:** Hyo Young Lee, Hyunwoo Oh, Eileen Laurel Yoon, Dae Won Jun

**Affiliations:** 1 Department of Internal Medicine, Hanyang University College of Medicine, Seoul 04763, Republic of Korea; catchhyong@gmail.com; 2 Department of Internal Medicine, Kangbuk Samsung Hospital, Sungkyunkwan University School of Medicine, Seoul 03181, Republic of Korea; asklepios1258@gmail.com; 3 Institute of Advanced BioConvergence, Hanyang Institute of Bioscience and Biotechnology, Hanyang University, Seoul 04763, Republic of Korea

**Keywords:** silymarin, chronic liver disease, MASLD, hepatic steatosis, liver fibrosis, noninvasive tests, systematic review

## Abstract

Background/Objectives: Silymarin is a widely used hepatoprotective agent; however, its effects on hepatic steatosis and fibrosis remain unclear. We systematically reviewed the effects of silymarin on liver biochemical parameters, fibrosis, steatosis, and cardiometabolic outcomes, as well as its safety, in chronic liver disease. Methods: PubMed, Embase, and the Cochrane Library were searched from inception to June 11, 2026. Randomized controlled trials (RCTs) and eligible comparative clinical studies that evaluated the use of oral silymarin were included. Risk of bias was evaluated using RoB 2 for randomized trials and ROBINS-I for non-randomized studies. Random-effects meta-analyses were performed for alanine aminotransferase (ALT) and aspartate aminotransferase (AST) in placebo-controlled RCTs of silymarin monotherapy and for liver stiffness measurement (LSM) in placebo-controlled RCTs, and the certainty of evidence was assessed using GRADE. Results: Forty-seven studies (37 RCTs and 10 non-randomized studies) met the inclusion criteria. Compared with placebo, silymarin monotherapy significantly reduced ALT (six trials; mean difference [MD], −7.25 IU/L; 95% confidence interval [CI], −11.60 to −2.89) and AST (four trials; MD, −5.36 IU/L; 95% CI, −8.45 to −2.27). In addition, an exploratory meta-analysis of three placebo-controlled RCTs showed a greater reduction in LSM with silymarin-based interventions than with placebo (MD, −0.60 kPa; 95% CI, −1.04 to −0.16). Conclusions: In placebo-controlled trials, silymarin monotherapy significantly reduced serum aminotransferase levels, and an exploratory meta-analysis of three trials also showed a reduction in liver stiffness with silymarin-based interventions. Silymarin was generally well tolerated. Well-designed long-term randomized controlled trials using validated noninvasive or histological fibrosis endpoints are warranted to determine whether these improvements translate into antifibrotic and clinically meaningful benefits.

## 1. Introduction

Chronic liver disease (CLD) is a major cause of morbidity and mortality worldwide. CLD accounts for more than 2 million deaths annually, representing approximately 4% of all deaths worldwide, with most liver-related deaths attributable to cirrhosis and hepatocellular carcinoma [1,2]. The most common etiologies include metabolic dysfunction-associated steatotic liver disease (MASLD), previously referred to as non-alcoholic fatty liver disease (NAFLD), hepatitis B virus (HBV), hepatitis C virus (HCV), and alcohol-related liver disease. While the incidence of HBV and HCV has decreased owing to widespread vaccination and antiviral treatment programs, the rising burden of obesity and metabolic syndrome has driven an alarming increase in MASLD, which now accounts for approximately 60% of the global incidence of CLD, particularly in developed countries [2]. Despite differences in underlying etiology, these conditions converge on a common pathological pathway: progressive hepatic fibrogenesis, which ultimately drives the transition to cirrhosis and liver-related mortality, regardless of the initial insult.

Hepatic fibrosis is characterized by excessive extracellular matrix protein deposition and scar tissue formation. Fibrosis is known to be the strongest histological predictor of morbidity and mortality in CLD [3,4], and as the condition advances, it can progress to cirrhosis and hepatocellular carcinoma [5]. Although advanced fibrosis was once considered largely irreversible, it is increasingly recognized that early-stage fibrosis can regress with appropriate interventions [6]. Therefore, the timely management of hepatic fibrosis before progression to advanced stages is critical for improving long-term outcomes. Despite the availability of disease-specific therapies for certain etiologies, effective pharmacological interventions that directly target hepatic fibrosis remain limited, representing an unmet medical need [7].

In metabolic dysfunction-associated steatohepatitis (MASH), MASH-specific pharmacological therapies have recently become available. Following its accelerated approval by the U.S. Food and Drug Administration (FDA) in 2024, resmetirom was endorsed in the EASL–EASD–EASO Clinical Practice Guidelines as a MASH-targeted therapy for adults with non-cirrhotic MASH and significant fibrosis, where locally approved and according to the label [8]. Semaglutide received accelerated FDA approval in 2025 for adults with MASH and moderate-to-advanced fibrosis (F2–F3), and the updated AASLD Practice Guidance provides recommendations for its clinical use in this population [9]. However, these therapies are limited to adults with non-cirrhotic MASH and moderate-to-advanced fibrosis, and no antifibrotic therapy has been approved across the broader spectrum of CLD.

Against this therapeutic landscape, silymarin has long been used as a hepatoprotective agent and has been investigated across a broad range of chronic liver diseases. Silymarin is a polyphenolic complex obtained from milk thistle (*Silybum marianum*) using organic or aqueous-organic solvents rather than aqueous extraction alone, owing to the low aqueous solubility of its flavonolignan constituents. Its hepatoprotective properties are attributed to multiple mechanisms including free radical scavenging, inhibition of lipid peroxidation, stabilization of hepatocyte membranes, enhancement of glutathione levels, and stimulation of protein synthesis. Silymarin also exhibits anti-inflammatory activity through the suppression of NF-κB and proinflammatory cytokines [10,11]. Beyond hepatoprotection, silymarin has also been investigated for metabolic, immunomodulatory, and iron-chelating effects in a variety of clinical and experimental settings [12].

In addition to its antioxidant and anti-inflammatory effects, silymarin has demonstrated significant antifibrotic potential in experimental models. Specifically, silymarin inhibits the activation of hepatic stellate cells (HSCs), the primary drivers of fibrogenesis, by downregulating transforming growth factor-beta 1 (TGF-β1) expression and reducing the production of α-smooth muscle actin and collagen type I [10,11]. These preclinical findings suggest that silymarin may modulate the fibrogenic pathway rather than merely providing indirect hepatoprotection.

The clinical efficacy of silymarin has been investigated in numerous randomized controlled trials (RCTs) across various CLD etiologies. However, most studies have primarily focused on liver enzyme levels and hepatic steatosis, whereas fibrosis-related outcomes have only been reported in a subset of trials. Previous systematic reviews and meta-analyses have largely confirmed the effects of silymarin on alanine aminotransferase (ALT) and aspartate aminotransferase (AST) levels [12,13]; however, a comprehensive synthesis encompassing both biochemical and fibrosis-specific outcomes, including imaging-, serum-, and histological markers, has not been performed. Therefore, this systematic review aimed to evaluate the efficacy and safety of silymarin across different CLD etiologies, with respect to liver biochemical, fibrosis-related, hepatic steatosis, and cardiometabolic outcomes.

## 2. Materials and Methods

### 2.1. Study Design

This systematic review was conducted in accordance with the Preferred Reporting Items for Systematic Reviews and Meta-Analyses (PRISMA) 2020 statement [14], and the study objectives were defined using the PICO/PECO framework (Population, Intervention/Exposure, Comparator, and Outcomes) (Table 1). The review protocol was registered in the International Prospective Register of Systematic Reviews (registration number: CRD420261452601). This systematic review used previously published data and therefore did not require institutional review board approval or informed consent.

### 2.2. Eligibility Criteria

Studies were selected according to the predefined eligibility criteria. We included randomized controlled trials and comparative non-randomized studies, including quasi-experimental, prospective controlled, and controlled observational studies. Noncomparative single-arm studies were excluded. Eligible studies evaluated oral silymarin or silybin-containing interventions administered for at least 4 weeks in adults aged ≥18 years with chronic liver disease. Eligible liver diseases included chronic hepatitis, cirrhosis, and steatotic liver diseases, including MASLD, NAFLD, MASH, NASH, and MAFLD. The eligible comparators included a placebo, standard treatment, no treatment, and the same background treatment without silymarin. Combination therapy studies were included if the incremental contribution of a silymarin-containing intervention could be reasonably evaluated against a concurrent comparator. These studies were analyzed separately from studies of pure silymarin monotherapy.

Outcomes used to determine study eligibility were prespecified. The primary outcomes were liver enzyme-related outcomes, including alanine aminotransferase (ALT), aspartate aminotransferase (AST), and gamma-glutamyl transferase (GGT). Secondary outcomes were fibrosis-related outcomes, comprising liver stiffness measurement (LSM), shear wave elastography (SWE), magnetic resonance elastography (MRE), the Fibrosis-4 index (FIB-4), the aspartate aminotransferase-to-platelet ratio index (APRI), the NAFLD fibrosis score (NFS), the enhanced liver fibrosis (ELF) test, and biopsy-assessed fibrosis stage; steatosis-related outcomes comprised fatty liver index (FLI), and fatty liver score (FLS). Baseline, post-intervention, and change-from-baseline values were eligible for extraction, and studies reporting at least one prespecified outcome were included.

We excluded studies primarily involving alcohol-related liver disease, alcohol consumption > 20 g/day, autoimmune hepatitis, Wilson’s disease, hemochromatosis, or alpha-1 antitrypsin deficiency, unless data for eligible participants could be separately extracted. Case reports and case series, reviews, editorials, protocols, and animal/in vitro studies were also excluded. Conference abstracts were excluded when they did not provide sufficient information to determine study eligibility. Studies that did not evaluate any of the prespecified outcomes were excluded.

### 2.3. Search Strategy

A comprehensive literature search was conducted using PubMed, Embase, and the Cochrane Library from database inception to June 11, 2026. The review followed the Preferred Reporting Items for Systematic Reviews and Meta-Analyses (PRISMA) guidelines (Supplementary Tables S1 and S2). The search strategy combined controlled vocabulary and free-text terms related to silymarin, chronic liver disease, and MASLD/NAFLD and was adapted to the indexing system and search syntax of each database. To enhance search sensitivity, supplementary searches were performed using expanded outcome terms, including the full terms and abbreviations for the fatty liver index (FLI) and fatty liver score (FLS), as well as terms related to histological fibrosis and fibrosis stage, while retaining the original population and intervention concepts and the search end date of 11 June 2026. The complete search strategies for all databases are provided in Supplementary Table S3. Reference lists of the included studies and relevant systematic reviews were also manually screened to identify additional eligible studies.

### 2.4. Study Selection

All retrieved references were imported into EndNote 2025 for reference management and initial duplicate removal. The remaining records were uploaded to Rayyan for further deduplication and screening. Two reviewers independently screened the titles and abstracts of all retrieved records according to predefined eligibility criteria. Full-text articles were obtained for eligible and potentially eligible studies. Two reviewers independently assessed the full-text articles and determined final study eligibility. Disagreements at either stage of screening were resolved through discussion. When consensus could not be reached, a third reviewer adjudicated the decision. Reasons for exclusion during the full-text screening stage were recorded. Additional duplicate records (*n* = 31) that were not detected during the identification stage because of inconsistent or incomplete bibliographic information were subsequently identified and removed during title and abstract screening. These records were recorded separately in the PRISMA flow diagram. The overall study selection process is summarized in the PRISMA flow diagram.

### 2.5. Data Extraction

Data were extracted into a standardized Microsoft Excel (Microsoft Corp.) template by one reviewer and cross-checked against original publications by a second reviewer. The extracted information included study details (author, publication year, country, study design, type of liver disease, and sample size), participant characteristics (age and sex), intervention characteristics, and outcome data (measurement method, unit, assessment time point, baseline and post-intervention values, and changes from baseline), with means and standard deviations preferentially extracted for continuous outcomes.

In addition to the prespecified outcomes used to determine study eligibility, other clinically relevant outcomes reported in eligible studies were extracted for narrative synthesis. These included the controlled attenuation parameter (CAP), imaging-based and histological steatosis, anthropometric, glycemic, lipid, and safety outcomes. These additional outcomes were not used to determine study eligibility.

### 2.6. Quality Assessment

The risk of bias in the included randomized controlled trials was independently assessed by two reviewers using the Cochrane Risk of Bias 2 (RoB 2) tool for specific outcomes, with each assessed result rated as low risk, some concerns, or high risk across five domains [15]. The risk of bias in the included non-randomized studies was assessed using the Risk of Bias in Non-randomized Studies of Interventions (ROBINS-I) tool, with each study rated as low, moderate, serious, or critical risk [16].

### 2.7. Statistical Analyses

Given the substantial clinical and methodological heterogeneity in intervention regimens, outcome measures, and follow-up durations, the study characteristics and outcomes are summarized descriptively. Narrative synthesis was performed separately for liver biochemical, fibrosis-related, hepatic steatosis, cardiometabolic, and safety outcomes, with between-group comparisons prioritized when available.

Meta-analyses were conducted for changes from baseline in alanine aminotransferase (ALT), aspartate aminotransferase (AST), and liver stiffness measurements (LSM). To isolate the effects of silymarin, the ALT and AST meta-analyses were restricted to randomized controlled trials (RCTs) comparing silymarin monotherapy with placebo, without concomitant lifestyle modifications or other interventions; these analyses were conducted post hoc. Because few trials assessed LSM, the LSM meta-analysis included all placebo-controlled RCTs with available data, including those in which identical background interventions were applied to both groups or a silymarin-containing combination was used. Studies were pooled when the mean change from baseline and its standard deviation (SD) could be obtained for each group. When only standard errors, 95% CIs, or *p*-values were reported, SDs were calculated using standard formulas; when only baseline and follow-up values were reported, the SD of the change was imputed assuming a correlation coefficient of 0.5 [Cochrane Handbook]. Studies that reported only medians with interquartile ranges, proportions of patients with normalized values, or values without measures of dispersion were described narratively. When a study included multiple silymarin dose groups sharing a common placebo group, the dose groups were combined into a single intervention group to avoid double-counting the control group.

Pooled effects were expressed as mean differences with 95% confidence intervals (CIs), using an inverse-variance random-effects model. Between-study variance (τ^2^) was estimated using the restricted maximum-likelihood method, and heterogeneity was assessed using Cochran’s Q test and the *I*^2^ statistic. Statistical analyses were performed using R version 4.5.3 and the meta-package version 8.5–0.

The certainty of evidence for the ALT, AST, and LSM meta-analyses was assessed using GRADE, and the results are presented in Supplementary Tables S4–S6.

## 3. Results

### 3.1. Search Results

**Figure 1:**
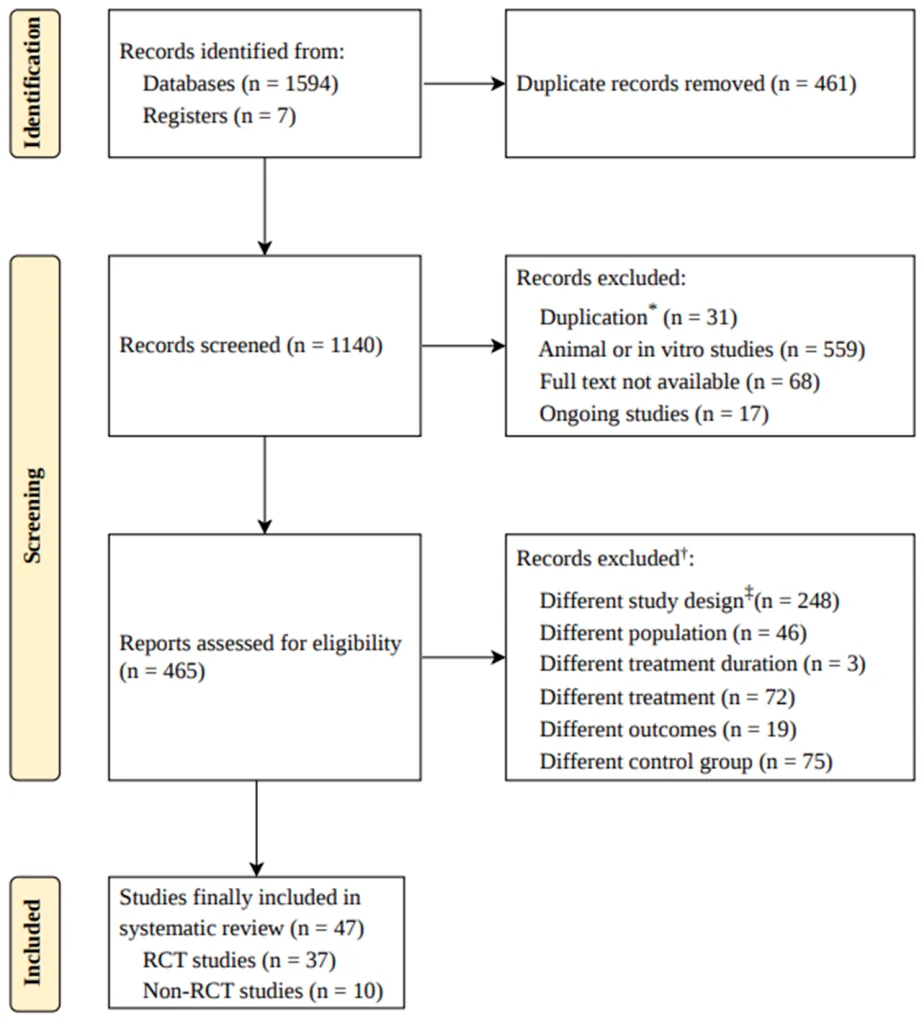
PRISMA diagram. * Duplicate records were removed using EndNote during the identification stage (*n* = 461). An additional 31 duplicates records that were not detected during the identification stage due to inconsistent or incomplete bibliographic information were subsequently removed. ^†^ The reasons for exclusion were not mutually exclusive, as some reports met more than one exclusion criterion. ^‡^ Different study designs included review articles, reports, protocols, trial registry records, and other ineligible publication types.

A total of 1601 records were identified, including 1594 retrieved through database searches and seven additional records identified through citation searches. After removing 461 duplicates, 1140 records underwent title and abstract screening. Of these, 675 records were excluded, with animal or in vitro studies being the most common reason for exclusion (*n* = 559). The full texts of 465 reports were assessed for eligibility, and 418 reports were excluded because they did not meet the predefined eligibility criteria. A total of 47 studies were ultimately included in the systematic review, comprising 37 randomized controlled trials and 10 non-randomized studies (Figure 1).

### 3.2. Characteristics of Included Studies

A total of 47 studies were included, comprising 37 randomized trials and 10 non-randomized studies (Table 2 and Table 3, Supplementary Tables S7 and S8). Of these, 35 studies enrolled patients with metabolic liver diseases, including non-alcoholic fatty liver disease, non-alcoholic steatohepatitis, metabolic dysfunction-associated steatotic liver disease, and hepatic steatosis. Among the remaining studies, 9 included patients with chronic hepatitis C, two included patients with chronic liver disease, and one study included patients with chronic active hepatitis. The interventions included silymarin, *Silybum marianum* extract, silybin, and silymarin- or silybin-containing complexes administered either alone or in combination with diet, exercise, lifestyle modification, antiviral therapy, or standard care. The control group received a placebo, no treatment, diet or lifestyle modification alone, standard care, antiviral therapy, or active comparators such as ursodeoxycholic acid or vitamin E. All silymarin preparations in the 47 included studies were derived from *Silybum marianum*, and no study evaluated chemically or microbially synthesized silymarin. Daily doses were reported in 45 studies and ranged from 70 to 2100 mg. Of these, 13 randomized studies evaluated doses within the approved daily range of 210–420 mg for standardized silymarin medicinal products. In the Republic of Korea, silymarin is authorized by the Ministry of Food and Drug Safety in 70 mg and 140 mg strengths administered three times daily, corresponding to total daily doses of 210 and 420 mg, respectively. Comparable dosing regimens are documented for nationally authorized medicinal products in Germany, including an initial daily dose of 420 mg and maintenance doses of 210 or 280 mg, depending on the product strength [17]. Neither authorization specifies a fixed treatment duration. ALT level was assessed in 46 studies and was the most frequently reported outcome. AST and GGT levels were assessed in 41 and 19 studies, respectively. Fibrosis-related outcomes were evaluated in 18 studies. These included liver stiffness measurements using FibroScan in 10 studies, FIB-4 index in 8 studies, histologic fibrosis stage in 6 studies, NFS in 6 studies, and APRI in 3 studies (Supplementary Table S9).

### 3.3. Risk-of-Bias Assessment

The risk of bias was assessed using the RoB 2 tool for randomized studies and the ROBINS-I tool for non-randomized studies [15,16]. RoB 2 assessments were conducted at the outcome-result level for the randomized trials. Risk-of-bias judgments varied across outcomes and studies, with concerns arising primarily from deviations from intended interventions, missing outcome data, and selection of the reported result. Detailed outcome-specific assessments are presented in Supplementary Figure S1. Among the 10 non-randomized studies, two were rated as having moderate, six serious, and two critical overall risk of bias, with the overall assessments driven by concerns related to confounding. Detailed assessments are presented in Supplementary Figure S2.

### 3.4. Liver Biochemical Outcomes

Liver biochemical outcomes were the most frequently reported endpoints (ALT, 46 studies; AST, 41 studies; GGT, 19 studies). Because the reported findings varied according to liver disease etiology, formulation, comparator, and whether within-group or between-group changes were considered, the findings of individual studies are described first, followed by meta-analyses of placebo-controlled RCTs of silymarin monotherapy for ALT and AST.

#### 3.4.1. Alanine Aminotransferase (ALT)

ALT reductions were reported across multiple RCTs, particularly among patients with NAFLD, MASLD, or NASH, in both monotherapy and combination-formulation studies; however, the strength of evidence varied depending on whether changes were significant between groups or only within the intervention group. ALT reductions were reported in monotherapy RCTs of varying durations, ranging from short-term (approximately 2 months) to longer-term (approximately 6 months) treatment, and across diverse dosing regimens [22,28,34,35,36,44,45,50,51]. Similarly, combination-formulation RCTs also reported ALT reductions, although interpretation of these findings was complicated by concomitant dietary interventions [18,30,54].

A smaller number of RCTs reported no significant differences in ALT levels between silymarin and control groups. In the NAFLD/MASLD population, these included a trial in which ALT reduction was significant only in the control group [21], where the trial did not meet its primary endpoint [24], and studies in which ALT remained unchanged despite significant improvement in liver stiffness [37], or steatosis grade on imaging [23]. In chronic hepatitis C, RCTs of silymarin monotherapy have reported no significant improvement in ALT levels in patients with ongoing HCV viremia [31,33,52]. ALT reductions were also reported in several non-randomized studies [55,56,58,61,63].

**Figure 2:**
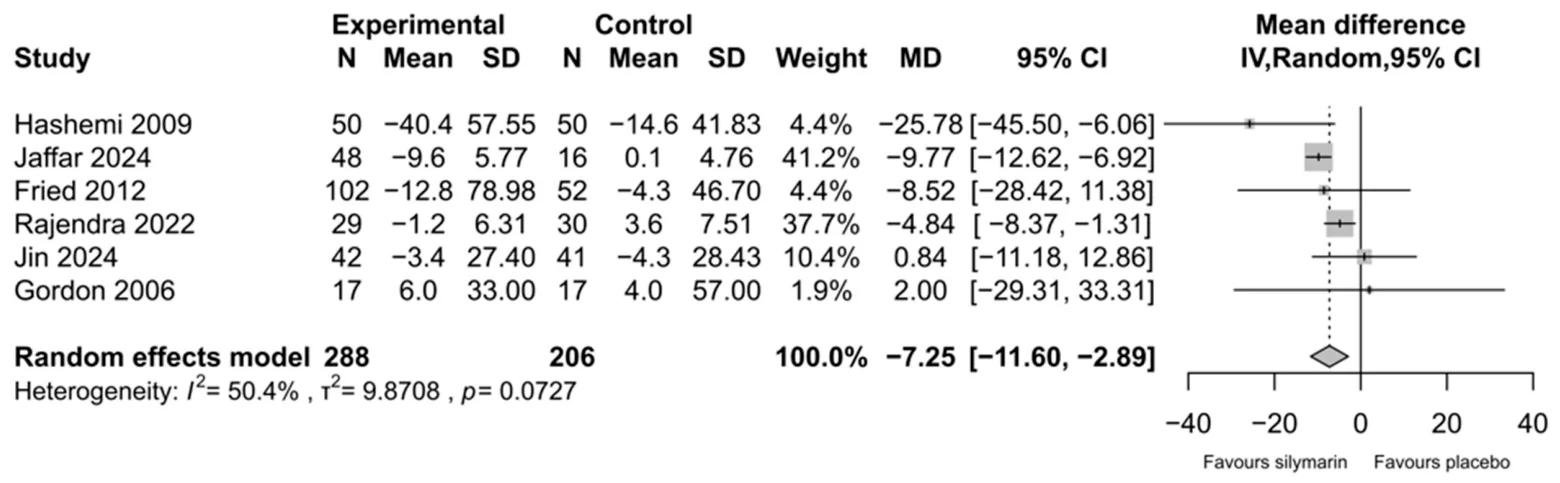
Forest plot of the effect of silymarin monotherapy versus placebo on change in ALT levels [31,33,35,36,37,48]. Abbreviations: CI, confidence interval; IV, inverse variance; MD, mean difference. The diamond represents the pooled estimate obtained using a random-effects model. Negative mean differences favor silymarin.

These differences across RCTs may be explained, at least in part, by differences in formulations, concomitant dietary or lifestyle interventions, and whether between-group or only within-group changes were reported. To estimate the effect attributable to silymarin itself, with minimal influence from concomitant interventions and combination formulations, a meta-analysis was performed restricted to placebo-controlled RCTs of silymarin monotherapy (six trials; 494 participants) [31,33,35,36,37,48]. The pooled analysis demonstrated a statistically significant reduction in ALT with silymarin compared with placebo (MD, −7.25 IU/L; 95% CI, −11.60 to −2.89; *I*^2^ = 50.4%) (Figure 2).

#### 3.4.2. Aspartate Aminotransferase (AST)

Reductions in AST levels were reported in several RCTs, particularly among patients with NAFLD, MASLD, or NASH, across both monotherapy and combination-formulation studies [22,34,35,36,44,45,46,49,50,51,54].

In contrast, no significant AST changes were reported in a small number of RCTs involving patients with NAFLD/MASLD [18,21,23,24,37]. In chronic hepatitis C RCTs, AST outcomes were largely not reported or showed no significant change. AST reductions were also reported in several non-randomized studies [56,58,61,63].

**Figure 3:**
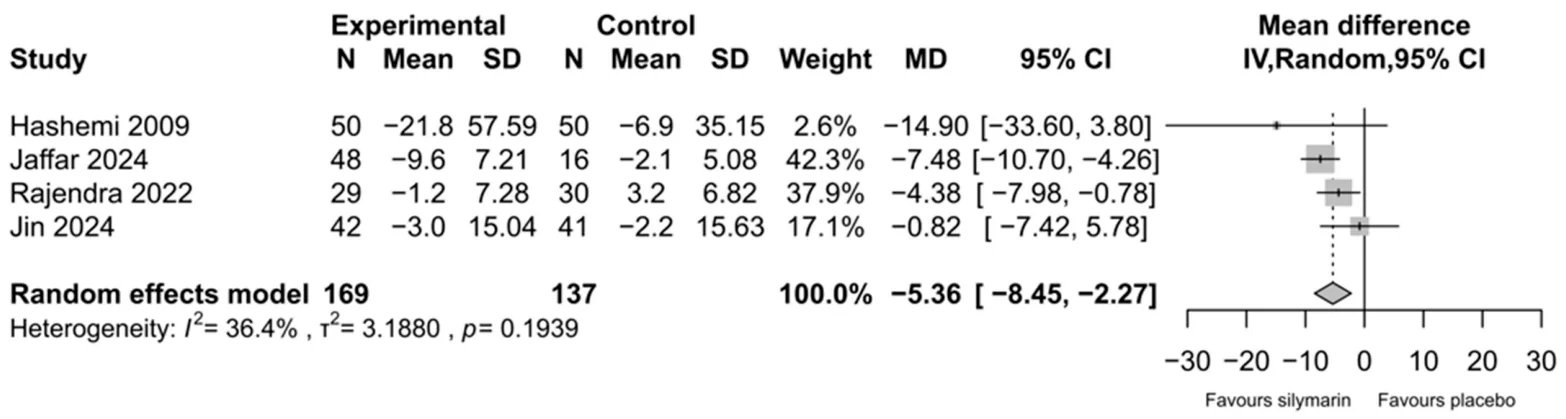
Forest plot of the effect of silymarin monotherapy versus placebo on change in AST levels [35,36,37,48]. Abbreviations: CI, confidence interval; IV, inverse variance; MD, mean difference. The diamond represents the pooled estimate obtained using a random-effects model. Negative mean differences favor silymarin.

As with ALT, AST findings varied across RCTs according to formulation, concomitant interventions, and whether between-group or only within-group changes were reported. Applying the same criteria, a meta-analysis of placebo-controlled RCTs of silymarin monotherapy (four trials; 306 participants) [35,36,37,48] showed a statistically significant reduction in AST compared with placebo (MD, −5.36 IU/L; 95% CI, −8.45 to −2.27; *I*^2^ = 36.4%) (Figure 3).

#### 3.4.3. Gamma-Glutamyl Transferase (GGT)

Fewer studies have reported GGT levels than ALT or AST levels. Reductions in GGT levels were reported in several RCTs [30,37,41], whereas one RCT found no significant changes [24]. GGT reductions were also reported in several non-randomized studies [57,58].

### 3.5. Fibrosis-Related Outcomes

Fibrosis-related outcomes were evaluated using liver stiffness measurements (LSM), serum-based noninvasive tests (NITs), and histological assessments.

#### 3.5.1. Liver Stiffness Measurements (LSM)

Several RCTs reported reductions in LSM. Jin et al. demonstrated a significant between-group reduction in mean LSM, whereas Wah Kheong et al. reported a significantly greater proportion of participants achieving a ≥30% reduction in LSM despite no significant between-group difference in mean LSM [26,37]. A significant LSM improvement was also reported in a combination-formulation RCT using an active comparator [25], whereas no significant effect was observed in an RCT using lifestyle intervention as a control [19]. In a study by Colletta et al., LSM decreased in both groups, with no significant additional benefit from silymarin [28]. In contrast, no significant FibroScan changes were reported in several RCTs, including a short-term (8-week) monotherapy RCT [46] and combination formulation RCTs [29,30,49]. Among non-randomized studies, no significant FibroScan changes have been reported [56].

**Figure 4:**
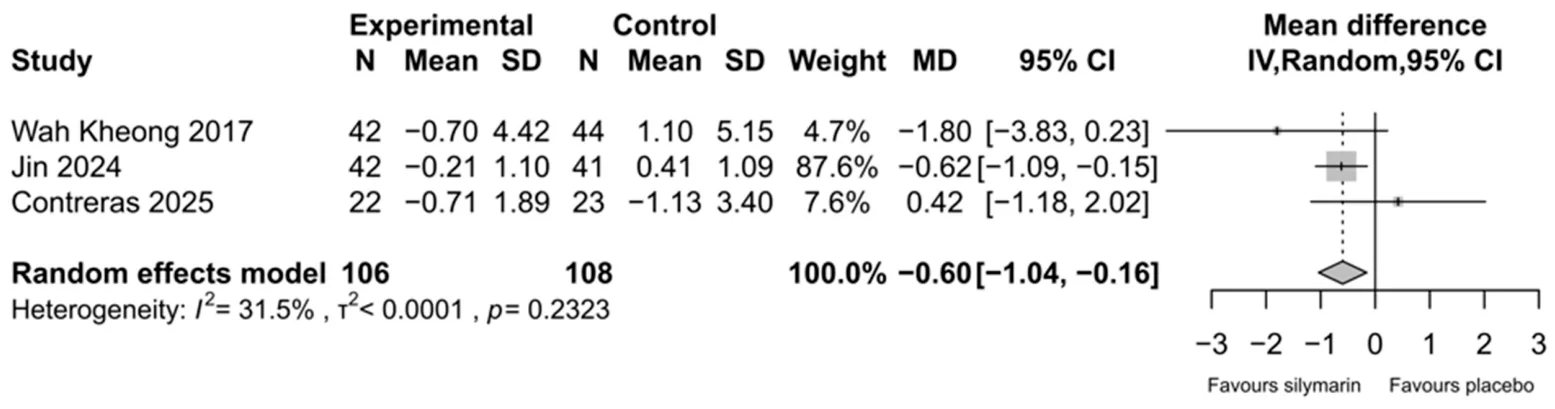
Forest plot of the effect of silymarin-based interventions versus placebo on change in liver stiffness measurement [26,29,37]. Abbreviations: CI, confidence interval; IV, inverse variance; LSM, liver stiffness measurement; MD, mean difference. The diamond represents the pooled estimate obtained using a random-effects model. Negative mean differences favor silymarin.

Because LSM findings varied across RCTs with different designs, comparators, and formulations, an exploratory meta-analysis was performed including the three placebo-controlled RCTs with available data (214 participants) [26,29,37], which showed a greater reduction in LSM with silymarin-based interventions than with placebo (MD, −0.60 kPa; 95% CI, −1.04 to −0.16; *I*^2^ = 31.5%) (Figure 4; Supplementary Table S6).

#### 3.5.2. Serum-Based Non-Invasive Tests (NITs)

Wah Kheong et al. reported that FIB-4, APRI, and NFS decreased from baseline in the silymarin group; however, the corresponding between-group differences were not statistically significant [26]. Likewise, Jin et al. and Shaker et al. reported no significant between-group differences in FIB-4 or APRI [37,49]. The NFS improved from baseline in several RCTs, although improvement was also observed in the control groups in some studies [21,26,28]. No significant change in NFS was observed in one short-term 8-week RCT [46]. Overall, serum-based NITs showed variable responses without consistent comparative effects.

#### 3.5.3. Histological Fibrosis and Activity Outcomes

Among the RCTs, paired liver biopsy for fibrosis stage evaluation has been employed in a limited number of studies. Histological outcomes were reported in two studies, which differed in the strength of the comparative evidence. Wah Kheong et al. reported a higher frequency of ≥1-stage fibrosis improvement with silymarin than with placebo [26]. Loguercio et al. reported a within-group histological improvement with a multicomponent silybin formulation, whereas a significant between-group effect was not established [41]. Navarro et al. found no significant superiority over placebo [47]. An additional RCT in chronic hepatitis C reported significant reductions in serum fibrosis markers (TGF-β, MMP-2, PIIINP) rather than histological staging [43].

Among non-randomized studies, an observational cohort of 1049 patients with hepatitis *C*-related advanced fibrosis or cirrhosis reported that baseline silymarin use was associated with reduced histological progression, although no effect on clinical outcomes was observed [60]. NAS has been evaluated in several biopsy-based RCTs, as described above. No significant NAS improvement was reported in two studies [26,47], whereas NAS improved only within the treatment group in one combination-formulation RCT [41].

### 3.6. Hepatic Steatosis Outcomes

Among the RCTs, reductions in FLI or steatosis scores were reported in several studies [18,21,27,48]. One study employed silymarin as an active comparator alongside a placebo arm [48], one compared silymarin plus vitamin E with a diet-only control [21], and two studies lacked a placebo arm, employing either a Mediterranean diet [18] or nutraceutical diet supplementation [27]. One RCT reported a significant between-group improvement in CAP at six months [30], and one short-term monotherapy RCT reported no significant change in CAP [37]. A combination-therapy RCT reported a significant within-group improvement in CAP, whereas between-group comparative results were not reported [29]. Among non-randomized studies, significant improvements in steatosis have been reported [57].

### 3.7. Cardiometabolic Outcomes

#### 3.7.1. Anthropometric Outcomes

Among the RCTs, improvements in BMI and waist circumference were reported in studies employing concurrent Mediterranean diet interventions [18,19], and one RCT reported significant improvement across all treatment groups, including the placebo group [22]. A significant BMI reduction was also reported in a short-term monotherapy RCT conducted in patients with morbid obesity [46]. In contrast, a few monotherapy RCTs have reported no significant changes in BMI, waist-to-hip ratio, or body fat percentage [37,44]. BMI reduction has been reported in non-randomized studies [61].

#### 3.7.2. Glycemic and Insulin-Resistance Outcomes

Improvements in glycemic control and insulin sensitivity have been reported in several RCTs. Significant improvements in insulin sensitivity have been observed in studies combining silymarin-containing formulations with a Mediterranean diet [18,19], whereas one study with a similar design reported a significant between-group reduction in HbA1c alone [28], and in a combination formulation compared with ursodeoxycholic acid [25]. One monotherapy RCT reported significant improvements in blood glucose and lipids [45], and another combination formulation RCT reported improvements in HOMA-IR and insulin [30]. One RCT reported significant improvement in insulin resistance in an HCV-positive subgroup [41]. Among the non-randomized studies, reductions in HbA1c were reported within the silymarin group in one study [61], whereas another study reported a significant between-group reduction in HbA1c [64]. In contrast, a rigorously controlled monotherapy RCT reported no significant effects on glycemic parameters [37].

#### 3.7.3. Lipid Profile Outcomes

Among the RCTs, significant improvements in lipid profiles have been reported in studies combining silymarin-containing formulations with a Mediterranean diet [18,19], as well as in a monotherapy RCT [45]. Among the non-randomized studies, reductions in total cholesterol, LDL, and triglycerides were reported in the silymarin group in one study [61]. However, such lipid changes have not been consistently demonstrated across trials. Another non-randomized study reported no significant between-group differences in lipid parameters [64], and a monotherapy RCT similarly revealed no significant alterations in circulating lipid levels [37].

### 3.8. Findings from Silymarin Monotherapy Studies

When studies evaluating silymarin monotherapy were considered separately, liver biochemical parameters were the most frequently assessed outcomes. Changes in ALT and AST were reported across monotherapy trials conducted in different chronic liver disease populations and treatment durations, although the magnitude and comparative effects varied according to the study population and design. In trials involving patients with active chronic hepatitis C infection, silymarin monotherapy did not demonstrate a clear comparative effect on aminotransferase levels. In meta-analyses restricted to placebo-controlled RCTs of silymarin monotherapy, silymarin significantly reduced ALT (MD, −7.25 IU/L; 95% CI, −11.60 to −2.89) and AST (MD, −5.36 IU/L; 95% CI, −8.45 to −2.27) compared with placebo.

Fibrosis-related outcomes were evaluated in a smaller number of placebo-controlled monotherapy trials. These studies included histological fibrosis assessment, liver stiffness measurement, and serum-based noninvasive tests. Changes in histological fibrosis or liver stiffness were reported in selected trials, whereas findings across different fibrosis-related measures within and between studies were heterogeneous.

Evidence for steatosis and cardiometabolic outcomes from monotherapy studies was more limited and heterogeneous. Overall, separating monotherapy studies from studies of combination formulations reduced the influence of concomitant active components or lifestyle interventions when considering findings potentially attributable to silymarin itself.

### 3.9. Outcomes in Studies Using Daily Dose of 210–420 mg

Thirteen studies evaluated daily doses of 210–420 mg across heterogeneous populations, formulations, study designs, and outcomes, with liver biochemical parameters being the most frequently reported outcomes. These studies demonstrate that the available evidence was not limited to higher-dose regimens.

### 3.10. Safety and Tolerability

Safety and tolerability data were reported in a subset of studies. Across studies that explicitly reported safety outcomes, silymarin was generally well tolerated, including trials evaluating doses of up to 2100 mg/day. No significant differences in adverse events between the silymarin and placebo groups have been reported in multiple RCTs [26,31,33,47], and the adverse events that occurred were not attributed to silymarin. Several RCTs reported no side effects and noted that silymarin was well tolerated [35,53]. Mild adverse events, predominantly transient gastrointestinal symptoms such as mild diarrhea, nausea, or abdominal discomfort, were reported in a minority of patients in both groups, with no significant between-group difference [29,31]. Among the non-randomized studies, one reported that the formulation was well tolerated, with no adverse events during the treatment period [57].

No serious adverse events attributed to silymarin were reported in any of the included studies. Treatment discontinuation owing to adverse events occurred in a small number of patients in one RCT, although these were not attributed to the study drug [41]. Overall, silymarin was well tolerated across the range of doses and treatment durations evaluated.

## 4. Discussion

### 4.1. Summary of Main Findings

In this systematic review of 47 studies, including 37 RCTs and 10 non-RCTs, the effects of silymarin on liver enzyme, fibrosis-related, steatosis, and cardiometabolic outcomes were evaluated.

In meta-analyses of placebo-controlled RCTs, silymarin monotherapy significantly reduced ALT and AST levels compared with placebo, and an exploratory meta-analysis of three placebo-controlled trials showed a greater reduction in LSM with silymarin-based interventions. Other fibrosis-related outcomes were evaluated in fewer studies, mainly those with longer treatment durations. Changes in cardiometabolic and anthropometric parameters were reported in some studies; however, it is difficult to distinguish the independent contribution of silymarin from that of concurrent lifestyle interventions. Given the close relationship between dietary habits, metabolic health, and liver-related outcomes, these findings should be interpreted in the context of concurrent dietary and lifestyle interventions. Silymarin should not be considered a substitute for appropriate dietary and lifestyle management. Silymarin was well tolerated and no serious treatment-related adverse events (SAEs) were reported.

### 4.2. Interpretation of Liver Biochemical Effects

Changes in liver biochemical parameters have been reported across multiple studies and have also been evaluated in previous systematic reviews and meta-analyses [12,13,65], although findings varied across individual trials. In the present review, meta-analyses restricted to placebo-controlled RCTs of silymarin monotherapy showed significant reductions in ALT (MD, −7.25 IU/L) and AST (MD, −5.36 IU/L), consistent with the direction of effect reported in previous meta-analyses [12,13,65]. Because these analyses were limited to placebo-controlled monotherapy trials, the pooled estimates are less likely to reflect the effects of concomitant active components or lifestyle interventions; the small number of contributing trials and differences in study populations and formulations are addressed in Section 4.9. The effects of silymarin on liver biochemical parameters may also vary according to the underlying liver disease; in patients with viral hepatitis and active viral replication, silymarin monotherapy showed limited efficacy against ALT, as discussed in Section 4.7.

Several studies have reported reductions in GGT [30,37,41]. However, GGT is a nonspecific biochemical marker influenced by multiple hepatic and extrahepatic processes [66]; therefore, changes in GGT cannot be attributed to a specific therapeutic mechanism or interpreted as evidence of fibrosis improvement.

### 4.3. Interpretation of Fibrosis-Related Outcomes

Among the three RCTs that used paired liver biopsies, Wah Kheong et al. demonstrated a significant between-group improvement in the fibrosis stage, whereas Loguercio et al. reported a within-group improvement with a multicomponent formulation. Navarro et al. found no significant improvement compared to placebo [26,41,47]. An additional observational study [60] reported that baseline silymarin use was associated with reduced histological progression in a hepatitis C cirrhosis cohort, although no effect on clinical outcomes was observed. Biopsy-based evidence is predominantly derived from NASH, HCV, and liver cirrhosis populations, and the histological response to silymarin in NAFLD/MASLD remains largely uncharacterized given the invasive nature of liver biopsy.

Owing to the limited feasibility of liver biopsy, most studies have relied on serum-based noninvasive tests (NITs) for fibrosis assessment. When individual fibrosis-related markers were evaluated separately, significant improvements were reported across several studies, although the specific markers that reached significance differed between studies. However, improvements have not been consistently observed across different fibrosis-related measures in individual studies. In study [37] (MASLD, silymarin monotherapy, 24 weeks), FibroScan showed a significant reduction in liver stiffness, whereas FIB-4 and APRI did not reach statistical significance. This discrepancy may reflect differences in the biological and statistical properties of the assessments. Specifically, because aminotransferase levels are incorporated into the calculation of FIB-4 and APRI, treatment-associated reductions in these enzymes may lower the scores even in the absence of fibrosis regression. Accordingly, changes in these indices are interpreted as indirect fibrosis-related signals rather than independent evidence of fibrosis regression, particularly in the absence of corroborating histological or imaging-based findings. However, LSM is also influenced by inflammation and other transient factors [67,68].

In placebo-controlled monotherapy trials, fibrosis-related outcomes were assessed using histology and LSM. In study [26], investigators reported a higher frequency of ≥ 1-stage fibrosis improvement and a higher proportion of participants achieving ≥ 30% LSM reduction with silymarin, whereas between-group differences in mean LSM and serum-based NITs were not statistically significant. In a study by Jin et al. [37], a significant between-group reduction in FibroScan-based LSM was observed. The LSM and histological fibrosis results in study [26] were rated as having some concerns, whereas the LSM result in study [37] had a low risk of bias. In an exploratory meta-analysis of three placebo-controlled trials, LSM decreased more with silymarin-based interventions than with placebo (MD, −0.60 kPa; 95% CI, −1.04 to −0.16), suggesting a possible effect on liver stiffness. An open-label trial without a placebo control [28], in which both groups received dietary intervention, was not included in this analysis. Given the small number of trials and the dominant weight of a single trial, this estimate should be interpreted with caution and confirmed in larger trials. Among studies evaluating LSM for ≥24 weeks, the magnitude and direction of LSM changes varied across interventions, comparators, and study designs (Supplementary Table S10).

Overall, fibrosis-related outcomes varied across studies, and differences in endpoints, interventions, comparators, and study designs limited direct cross-study comparisons.

### 4.4. Interpretation of Hepatic Steatosis Outcomes

Changes in non-invasive steatosis indices were reported across several studies [18,21,27,29,48,57], broadly consistent with previous systematic reviews evaluating the effects of silymarin on steatosis-related outcomes [69]. However, the frequent use of concomitant lifestyle interventions or combination formulations complicates attribution of the findings to silymarin alone. The relatively early improvement in the FLI may be compatible with the faster response to steatosis than to fibrosis [70], although this remains an exploratory interpretation.

### 4.5. Interpretation of Cardiometabolic Effects

Cardiometabolic findings were mixed and appeared to depend partly on concomitant lifestyle interventions and formulations. Although improvements in glycemic, lipid, and anthropometric parameters have been reported in several studies, these findings concentrated on trials largely incorporating lifestyle modification [18,19,28], combination formulations [18,19,25,41], or non-randomized designs [61,64]. Placebo-controlled monotherapy studies have yielded conflicting results [37,45], consistent with previous meta-analyses that showed no clear effects on BMI, body weight, or waist circumference [69,71].

### 4.6. Treatment Duration-Dependent Effects of Silymarin: Liver Enzymes Versus Fibrosis

Interestingly, significant reductions in ALT and AST were reported in studies with treatment durations of three months or less [36,46,50,54]. This early biochemical response was also demonstrated in the findings of a recent meta-analysis of 55 RCTs, which demonstrated that silymarin treatment for ≤2 months was associated with even greater reductions in ALT and AST compared to longer treatment durations [12].

Fibrosis-related improvements were reported in selected studies with a treatment duration of approximately 6 months or longer, whereas shorter studies generally did not demonstrate clear changes in fibrosis-related endpoints. However, these observations are descriptive and do not establish a duration-response relationship. For instance, short-term studies of 8–12 weeks did not show clear changes in LSM or serum-based NITs [46,56], whereas the three trials pooled in the LSM meta-analysis, which showed a greater reduction in LSM with silymarin-based interventions, had treatment durations of 24–48 weeks [26,29,37]. A treatment duration of at least 1–2 years is recommended in NASH clinical trials to demonstrate fibrosis improvement [72], and the FDA guidelines also recommend a follow-up period of at least 12–18 months to evaluate the histological endpoints [73]. Mechanistically, fibrosis reversal involves the degradation and remodeling of the extracellular matrix, a process that requires substantial time [74]. The longer treatment duration associated with fibrosis-related improvement may reflect the slower time course of structural changes in the liver compared with biochemical responses. In contrast, serum liver enzymes may respond earlier to changes in hepatocellular injury or inflammation.

Thus, the observed pattern may reflect different time courses of biochemical and structural responses, with aminotransferase changes emerging earlier than fibrosis-related changes. As relatively few long-term monotherapy studies are available, this duration-response hypothesis should be evaluated prospectively in trials with standardized fibrosis endpoints.

### 4.7. Effects in Chronic Hepatitis C

The studies of viral hepatitis discussed in this section involved patients with chronic hepatitis C. In patients with active viral replication, silymarin monotherapy did not clearly reduce ALT levels in RCTs published between 2004 and 2012 [31,33,52], in line with a previous review [75]. In contrast, ALT levels improved when silymarin was used in combination with antiviral therapy [20,43], although an additional effect over antiviral therapy alone was not clearly established, and improvements in fibrosis markers were reported after viral suppression [62]. Because these studies were few in number and heterogeneous in design, and limited to chronic hepatitis C, these findings may not be generalizable to other forms of viral hepatitis.

Limited oral bioavailability, compared with the responses reported with high-dose intravenous silibinin, may partly explain the findings during active viremia [76]; this hypothesis, as well as the potential modifying role of viral suppression, warrants further evaluation.

### 4.8. Clinical and Research Implications

From a clinical perspective, the meta-analyses of placebo-controlled monotherapy RCTs provide quantitative evidence that silymarin reduces ALT and AST levels. Studies evaluating daily doses of 210–420 mg, the approved daily range for standardized silymarin medicinal products, were also included (Section 3.9), indicating that the available evidence was not limited to higher-dose regimens.

Histological and LSM changes were also reported in selected placebo-controlled monotherapy trials, providing findings of potential relevance to fibrosis-related outcomes independent of concomitant treatment. As fibrosis is a major determinant of long-term prognosis, these findings are potentially clinically relevant but should be interpreted in the context of the limited and heterogeneous evidence across fibrosis-related endpoints.

Clinically, the reduction in aminotransferase levels is supported by quantitative synthesis, and silymarin was generally well tolerated. Evidence for fibrosis-related outcomes remains more limited, and long-term controlled trials are needed to determine whether these biochemical effects translate into improvements in fibrosis and liver-related outcomes.

Given the substantial heterogeneity in formulations and study designs across the existing literature, interpretation of treatment effects should distinguish silymarin monotherapy from combination formulations and place greater weight on controlled studies using clinically validated endpoints. The separate consideration of silymarin monotherapy is particularly useful for evaluating findings that may be attributable to silymarin itself, because studies of combination formulations and concomitant lifestyle or pharmacological interventions do not allow the independent contribution of silymarin to be readily distinguished. However, this analytical distinction should not be interpreted as implying that monotherapy necessarily represents the preferred clinical use of silymarin, as its clinical role may vary according to the underlying liver disease, treatment setting, and locally approved indications [77].

### 4.9. Strengths and Limitations

To the best of our knowledge, the present study is one of the most comprehensive systematic reviews of silymarin. Unlike earlier SRs and meta-analyses that focused primarily on liver enzyme (ALT/AST) improvement, fibrosis-related outcomes were systematically classified into imaging-based, serum-based, and histological markers, and analyzed independently. The inclusion of a substantial number of monotherapy studies allowed for a more direct evaluation of findings associated with silymarin, separate from those of combination formulations, and the incorporation of long-term RCTs provided evidence relevant to fibrosis evaluation. In addition, meta-analyses restricted to placebo-controlled monotherapy RCTs provided quantitative estimates of effects attributable to silymarin itself.

This study had several limitations. First, substantial clinical and methodological heterogeneity across target diseases, formulations, doses, comparators, treatment durations, and outcome definitions limited the quantitative synthesis to ALT, AST, and LSM, and each meta-analysis included a small number of trials; in particular, most of the weight in the LSM analysis was contributed by a single trial. Findings for the other outcomes were synthesized narratively and should be interpreted descriptively. Second, individual fibrosis endpoints, particularly paired histology, were evaluated in relatively few studies, and several reported changes were based on within-group rather than between-group comparisons. Such within-group changes cannot establish a treatment effect and may be influenced by confounding factors. Third, the inclusion of small, open-label, single-center, and non-randomized studies warrants cautious interpretation of the findings because of their susceptibility to confounding and other sources of bias. Fourth, potential effect modification by age, sex, and baseline disease severity could not be evaluated because these variables were inconsistently reported across studies. Finally, adverse event reporting was not standardized, and publication bias could not be formally assessed because fewer than 10 studies were available for each meta-analysis.

## 5. Conclusions

This systematic review evaluated the liver biochemical, fibrosis-related, steatosis, and cardiometabolic outcomes of silymarin-based interventions in chronic liver disease. In meta-analyses of placebo-controlled RCTs, silymarin monotherapy significantly reduced ALT and AST levels, suggesting a potential benefit on liver biochemical parameters. These findings were consistent with the direction of effect reported in previous meta-analyses. An exploratory meta-analysis of three placebo-controlled trials also showed a greater reduction in liver stiffness with silymarin-based interventions than with placebo. Silymarin was generally well tolerated. Long-term, adequately powered trials with validated fibrosis endpoints are warranted to determine whether these biochemical and elastographic changes translate into clinically meaningful benefits in liver fibrosis.

## Figures and Tables

**Table 1:** PICO/PECO framework for study selection.

Variable	Definition
Population	Adults aged ≥18 years with chronic liver disease, including chronic hepatitis, cirrhosis, MASLD/NAFLD, MASH/NASH, and MAFLD.
Interventions	Oral silymarin or silybin-containing interventions, administered alone or in combination for ≥4 weeks.
Comparators	Placebo, standard treatment, no treatment, or identical background treatment without silymarin.
Outcomes	Primary: Liver enzymes (ALT, AST, GGT) Secondary: Fibrosis-related outcomes (LSM, SWE, MRE, serum-based NITs (FIB-4, APRI, NFS, ELF), and biopsy-assessed fibrosis stage) and steatosis-related outcomes (FLI, FLS).

Abbreviations: ALT, alanine aminotransferase; APRI, aspartate aminotransferase-to-platelet ratio index; AST, aspartate aminotransferase; FIB-4, Fibrosis-4 index; FLI, fatty liver index; GGT, gamma-glutamyl transferase; LSM, liver stiffness measurement; MAFLD, metabolic dysfunction-associated fatty liver disease; MASH, metabolic dysfunction-associated steatohepatitis; MASLD, metabolic dysfunction-associated steatotic liver disease; NAFLD, nonalcoholic fatty liver disease; NASH, nonalcoholic steatohepatitis; NFS, NAFLD fibrosis score; NITs, non-invasive tests. Studies reporting at least one pre-specified outcome were eligible for inclusion. Fibrosis outcomes were categorized as imaging-based, serum-based noninvasive tests (NITs), and histological assessment.

**Table 2:** Characteristics of the included randomized controlled trials (*n* = 37).

Study	Design	N (E/C)	Disease	Dose (mg/day)	Formulation	Comparator	Duration (weeks)	Outcomes
Abenavoli et al. (2015) [18]	3-arm RCT	10/10	NAFLD	188	Combination	Diet	24	ALT, AST, GGT, FLI
Abenavoli et al. (2015) [18]	3-arm RCT	10/10	NAFLD	188	Combination	None	24	ALT, AST, GGT, FLI
Abenavoli et al. (2017) [19]	3-arm RCT	20/20	NAFLD	240	Combination	Diet and exercise	24	ALT, AST, GGT, FLI LSM
Abenavoli et al. (2017) [19]	3-arm RCT	20/10	NAFLD	240	Combination	None	24	ALT, AST, GGT, FLI LSM
Ahmed et al. (2022) [20]	OL-RCT	15/15	CHC	400	Single	Antiviral therapy	8	ALT, AST, GGT
Aller et al. (2015) [21]	Pilot RCT	18/18	NAFLD	540.3 ^†^	Combination	Diet and exercise	12	ALT, AST, GGT, FLI, NFS
Anushiravani et al. (2019) [22]	DB-PC RCT	30/30	NAFLD	140	Single	Placebo *	12	ALT, AST
Ataee et al. (2021) [23]	TB-PC RCT	30/30	NAFLD	70	Combination	Placebo + diet	12	ALT, AST, LSM
Cerletti et al. (2020) [24]	MC DB-RCT	55/58	NAFLD	150	Combination	Control capsules	13	ALT, AST, GGT
Chakrabarty et al. (2021) [25]	DB Active RCT	88	NAFLD	420	Combination	UDCA *	26	ALT, LSM
**Wah Kheong** et al., (2017) [26]	DB-PC RCT	49/50	NASH	2100	Single	Placebo *	48	ALT, AST, GGT APRI, FIB-4, NFS, Histological fibrosis, LSM
Chiurazzi et al. (2022) [27]	RCT	36/32	NAFLD	≥200	Combination	Diet	12	ALT, AST
Colletta et al. (2020) [28]	OL Active RCT	108/104	NAFLD	420	Single	Diet	26	ALT, AST, FIB-4, NFS, LSM
Cano Contreras et al. (2025) [29]	DB-PC RCT	25/25	MASLD	600 ^†^	Combination	Placebo + diet	24	ALT, AST, GGT, LSM
Federico et al. (2019) [30]	NT-RCT	60/30	NAFLD	606 ^†^	Combination	None	26	ALT, AST, GGT, FIB-4, NFS, LSM
Fried et al. (2012) [31]	MC DB-PC RCT	50/52	CHC	1260	Single	Placebo	24	ALT
Fried et al. (2012) [31]	MC DB-PC RCT	52/52	CHC	2100	Single	Placebo	24	ALT
Gabbay et al. (2007) [32]	Phase II DB-PC RCT	25/25	CHC	750	Combination	Placebo + intravenous saline	24	ALT, AST
Gabbay et al. (2007) [32]	Phase II DB-PC RCT	25/25	CHC	750	Combination	Placebo	24	ALT, AST
Gordon et al. (2006) [33]	Cross-over RCT	24	CHC	960	Single	Placebo	12	ALT, GGT
Gordon et al. (2006) [33]	Cross-over RCT	24	CHC	480	Single	Placebo	12	ALT, GGT
Hajaghamohammadi et al. (2008) [34]	PC-RCT	25/25	NAFLD	140	Single	Placebo	8	ALT, AST
Hashemi et al. (2009) [35]	PC-RCT	50/50	NAFLD/NASH	280	Single	Placebo	24	ALT, AST
Jaffar et al. (2024) [36]	4-arm RCT	17/17	NAFLD	200	Single	Placebo	12.9	ALT, AST
Jaffar et al. (2024) [36]	4-arm RCT	18/17	NAFLD	400	Single	Placebo	12.9	ALT, AST
Jaffar et al. (2024) [36]	4-arm RCT	18/17	NAFLD	300	Single	Placebo	12.9	ALT, AST
Jin et al. (2024) [37]	DB-PC RCT	42/41	MASLD	103.2	Single	Placebo	24	ALT, AST, GGT FIB-4, APRI, LSM
Kaab Omeir (2025) [38]	4-arm RCT	10/10	NAFLD	140	Single	None	8	ALT, AST
Kaab Omeir (2025) [38]	4-arm RCT	10/10	NAFLD	140	Single	Exercise	8	ALT, AST
Li et al. (2024) [39]	TB-PC RCT	60/61	NAFLD	138	Combination	Placebo	24	ALT, AST, GGT FIB-4, APRI, FLI, NFS
Lirussi et al. (1989) [40]	Active RCT	9/9	Chronic active hepatitis	420	Single	UDCA	24	ALT, AST, GGT
Loguercio et al. (2012) [41]	Phase III MC DB-PC RCT	91/88	NAFLD	188	Combination	Placebo *	52	ALT, AST, GGT
Loguercio et al. (2012) [41]	Phase III MC DB-PC RCT	91/88	NAFLD	188	Combination	Placebo *	52	ALT, AST, GGT Histological fibrosis
Mai et al. (2025) [42]	DB-PC 3-arm RCT	32/31	NAFLD	163.2	Combination	Placebo *	12	ALT, AST, GGT
Mai et al. (2025) [42]	DB-PC 3-arm RCT	31/31	NAFLD	163.2	Single	Placebo *	12	ALT, AST, GGT
Malaguarnera et al. (2015) [43]	DB-PC RCT	32/32	CHC	94	Combination	Placebo + antiviral therapy	52	ALT, AST, Histological fibrosis
Masoodi et al. (2013) [44]	DB-PC RCT	50/50	NASH	280	Single	Placebo *	12	ALT, AST
Memon et al. (2015) [45]	DB-PC RCT	33/31	NAFLD	280	Single	Placebo *	12	ALT, AST
Mirhashemi et al. (2022) [46]	DB-PC RCT	30/30	NAFLD	560	Single	Placebo *	8	ALT, AST, FIB-4, NFS, Histological fibrosis
Navarro et al. (2019) [47]	Phase II MC DB-PC RCT	26/25	NASH	1260	Single	Placebo	48–50	ALT, AST, Histological fibrosis
Navarro et al. (2019) [47]	Phase II MC DB-PC RCT	27/25	NASH	2100	Single	Placebo	48–50	ALT, AST, Histological fibrosis
Rajendra et al. (2022) [48]	DB-PC 3-arm RCT	30/30	NAFLD	320	Single	Placebo	12	ALT, AST, GGT, FLI
Shaker et al. (2025) [49]	Phase II MC DB-PC RCT	49/48	Hepatic steatosis	600 ^†^	Combination	Placebo	36	ALT, AST, FIB-4, LSM
Shaker et al. (2025) [49]	Phase II MC DB-PC RCT	49/48	Hepatic steatosis	300 ^†^	Combination	Placebo	36	ALT, AST, FIB-4, LSM
Solhi et al. (2014) [50]	PC-RCT	40/40	NASH	210	Single	Placebo *	8	ALT, AST
Taghvaei et al. (2013) [51]	DB-PC RCT	21/20	NAFLD/NASH	280	Single	Placebo *	24	ALT, AST
Tanamly et al. (2004) [52]	DB Active RCT	88/89	CHC	373.5	Single	Multivitamin	52	ALT
Torres et al. (2004) [53]	Pilot RCT	17/17	CHC	480 ^†^	Single	None	4	ALT, AST
Zojaji et al. (2022) [54]	DB-RCT	40/20	NAFLD	480 ^†^	Combination	Diet and exercise	8	ALT, AST

Abbreviations: ALT, alanine aminotransferase; APRI, aspartate aminotransferase-to-platelet ratio index; AST, aspartate aminotransferase; C, control group; CHC, chronic hepatitis C; DB, double-blind; E, experimental group; FIB-4, Fibrosis-4 index; FLI, fatty liver index; GGT, gamma-glutamyl transferase; LSM, liver stiffness measurement; MASLD, metabolic dysfunction-associated steatotic liver disease; MC, multicenter; NAFLD, nonalcoholic fatty liver disease; NASH, nonalcoholic steatohepatitis; NFS, NAFLD fibrosis score; NT, no-treatment-controlled; OL, open-label; PC, placebo-controlled; RCT, randomized controlled trial; TB, triple-blind; UDCA, ursodeoxycholic acid. Studies with multiple eligible intervention arms were presented as separate comparisons. The combination formulations contained silymarin or silybin, together with one or more additional active components. * Combined with lifestyle modifications, including diet and/or exercise. ^†^ Exact silymarin/silybin content was not reported; doses refer to the *S. marianum* material or preparation.

**Table 3:** Characteristics of the included non-randomized comparative studies (*n* = 10).

Study	Design	N (E/C)	Disease	Dose (mg/day)	Formulation	Comparator	Duration (months)	Outcomes
Akhondi-Meybodi et al. (2020) [55]	Quasi-experimental, non-randomized	40/40	NAFLD	280	Single	Vitamin E + diet	4	ALT
Amjad et al. (2024) [56]	Non-randomized trial	73/68	Chronic liver disease	400	Single	Standard care	3	ALT, AST, LSM
Curcio et al. (2020) [57]	Controlled observational	41/40	NAFLD	280	Combination	Lifestyle modification	3	ALT, AST, GGT
Falasca et al. (2008) [58]	Observational controlled study	30/10	CHC	188	Combination	Lifestyle modification	3	ALT, AST, GGT
Federico et al. (2017) [59]	Prospective	17/10	NASH	NR	Combination	Placebo	12	ALT, AST, GGT
Freedman et al. (2011) [60]	Prospective	170/701	CHC	NR	Single	No silymarin	35	Histological fibrosis
Hasan (2024) [61]	Quasi-experimental	30/30	NAFLD	1500	Single	Standard care	6	ALT, AST
Rewisha et al. (2021) [62]	Prospective controlled	150/150	Chronic liver disease	420	Single	Antiviral	9	ALT, AST, FIB-4
Shaikh et al. (2021) [63]	Case–control	100/100	NAFLD	200	Single	Placebo *	3	ALT, AST
Sorrentino et al. (2015) [64]	Controlled observational	43/35	NAFLD	250	Combination	Diet and exercise	3	ALT, AST, GGT

Abbreviations: ALT, alanine aminotransferase; AST, aspartate aminotransferase; C, control group; E, experimental group; FIB-4, Fibrosis-4 index; GGT, gamma-glutamyl transferase; LSM, liver stiffness; NAFLD, nonalcoholic fatty liver disease; CHC, chronic hepatitis C; NASH, nonalcoholic steatohepatitis; NR, not reported. * Combined with lifestyle modifications, including diet and/or exercise.

## Data Availability

All data analyzed in this systematic review were derived from published studies cited in the article and its Supplementary Materials. The data supporting these findings are available from the corresponding authors upon request.

## Supplementary Materials

The following supporting information can be downloaded at: https://www.mdpi.com/article/10.3390/nu18213685/s1. Table S1: PRISMA 2020 checklist; Table S2: PRISMA 2020 abstract checklist; Table S3: Search strategies for the electronic databases; Table S4: GRADE assessment of the certainty of evidence for change in ALT; Table S5: GRADE assessment of the certainty of evidence for change in AST; Table S6: GRADE assessment of the certainty of evidence for change in liver stiffness measurement; Table S7: Detailed characteristics of the included randomized controlled trials (n = 37); Table S8: Detailed characteristics of the included non-randomized comparative studies (n = 10); Table S9: Outcomes assessed in the included studies; Table S10: Study characteristics and liver stiffness measurement findings in trials with treatment durations of ≥24 weeks; Figure S1: Outcome-specific risk-of-bias assessment of randomized controlled trials using the Cochrane Risk of Bias 2 tool. Assessments are presented separately for (a) ALT, (b) AST, (c) GGT, (d) FIB-4, (e) LSM, (f) APRI, (g) NFS, (h) histological fibrosis, and (i) FLI. Green indicates low risk, yellow indicates some concerns, and red indicates high risk of bias; Figure S2: Risk-of-bias assessment of the included non-randomized studies using the ROBINS-I tool. Judgments were categorized as low, moderate, serious, critical, or no information, according to the ROBINS-I framework.

## Abbreviations

ALT	Alanine aminotransferase
APRI	Aspartate aminotransferase-to-platelet ratio index
AST	Aspartate aminotransferase
BMI	Body mass index
C	Control group
CAP	Controlled attenuation parameter
CHC	Chronic hepatitis C
CI	Confidence interval
CLD	Chronic liver disease
DB	Double-blind
E	Experimental group
ELF	Enhanced liver fibrosis test
FIB-4	Fibrosis-4 index
FLI	Fatty liver index
FLS	Fatty liver score
GGT	Gamma-glutamyl transferase
GRADE	Grading of Recommendations Assessment, Development and Evaluation
HbA1c	Glycated hemoglobin
HBV	Hepatitis B virus
HCV	Hepatitis C virus
HDL-C	High-density lipoprotein cholesterol
HOMA-IR	Homeostatic model assessment of insulin resistance
HSC	Hepatic stellate cell
LDL-C	Low-density lipoprotein cholesterol
LSM	Liver stiffness measurement
MAFLD	Metabolic dysfunction-associated fatty liver disease
MASH	Metabolic dysfunction-associated steatohepatitis
MASLD	Metabolic dysfunction-associated steatotic liver disease
MC	Multicenter
MRE	Magnetic resonance elastography
N	Sample size
NAFLD	Nonalcoholic fatty liver disease
NASH	Nonalcoholic steatohepatitis
NFS	Nonalcoholic fatty liver disease fibrosis score
NIT	Noninvasive test
NITs	Noninvasive tests
NR	Not reported
NT	No-treatment-controlled
OL	Open-label
PC	Placebo-controlled
PECO	Population, exposure, comparator, and outcome
PICO	Population, intervention, comparator, and outcome
PRISMA	Preferred Reporting Items for Systematic Reviews and Meta-Analyses
Q1	First quartile
Q2	Second quartile
Q3	Third quartile
Q4	Fourth quartile
RCT	Randomized controlled trial
ROB 2	Risk of Bias 2
ROBINS-I	Risk of Bias in Non-randomized Studies of Interventions
SAE	Serious adverse event
SWE	Shear wave elastography
TB	Triple-blind
TGF-β1	Transforming growth factor beta 1
UDCA	Ursodeoxycholic acid
