# Supplementary material for: Silymarin-Based Therapies in Chronic Liver Disease: A Systematic Review of Biochemical, Cardiometabolic, Steatosis, and Fibrosis-Related Outcomes
Source: Nutrients. 2026 Nov 6;18(21):3685. doi: 10.3390/nu18213685 (PMC13765275; doi:10.3390/nu18213685)
Supplement: Supplementary file 1 [file nutrients-18-03685-s001.zip › nutrients-4523389-supplementary.pdf]

SUPPLEMENTARY MATERIALS

Table S1. PRISMA 2020 checklist

Section and Topic	Item #	Checklist item	Location where item is reported
**TITLE**			
Title	1	Identify the report as a systematic review.	Title
**ABSTRACT**			
Abstract	2	See the PRISMA 2020 for Abstracts checklist.	Abstract; Supplementary Table S2
**INTRODUCTION**			
Rationale	3	Describe the rationale for the review in the context of existing knowledge.	Section 1. Introduction
Objectives	4	Provide an explicit statement of the objective(s) or question(s) the review addresses.	Section 1. Introduction (final paragraph)
**METHODS**			
Eligibility criteria	5	Specify the inclusion and exclusion criteria for the review and how studies were grouped for the syntheses.	Section 2.2. Eligibility Criteria; Table 1
Information sources	6	Specify all databases, registers, websites, organisations, reference lists and other sources searched or consulted to identify studies. Specify the date when each source was last searched or consulted.	Section 2.3. Search Strategy
Search strategy	7	Present the full search strategies for all databases, registers and websites, including any filters and limits used.	Section 2.3. Search Strategy; Supplementary Table S3
Selection process	8	Specify the methods used to decide whether a study met the inclusion criteria of the review, including how many reviewers screened each record and each report retrieved, whether they worked independently, and if applicable, details of automation tools used in the process.	Section 2.4. Study Selection
Data collection process	9	Specify the methods used to collect data from reports, including how many reviewers collected data from each report, whether they worked independently, any processes for obtaining or confirming data from study investigators, and if applicable, details of automation tools used in the process.	Section 2.5. Data Extraction
Data items	10a	List and define all outcomes for which data were sought. Specify whether all results that were compatible with each outcome domain in each study were sought (e.g. for all measures, time points, analyses), and if not, the methods used to decide which results to collect.	Sections 2.2 and 2.5; Table 1
	10b	List and define all other variables for which data were sought (e.g. participant and intervention characteristics, funding sources). Describe any assumptions made about any missing or unclear information.	Section 2.5. Data Extraction; Supplementary Tables S7 and S8
Study risk of bias assessment	11	Specify the methods used to assess risk of bias in the included studies, including details of the tool(s) used, how many reviewers assessed each study and whether they worked independently, and if applicable, details of automation tools used in the process.	Section 2.6. Quality Assessment
Effect measures	12	Specify for each outcome the effect measure(s) (e.g. risk ratio, mean difference) used in the synthesis or presentation of results.	Section 2.7. Statistical Analyses

Synthesis methods	13a	Describe the processes used to decide which studies were eligible for each synthesis (e.g. tabulating the study intervention characteristics and comparing against the planned groups for each synthesis (item #5)).	Sections 2.2 and 2.7
	13b	Describe any methods required to prepare the data for presentation or synthesis, such as handling of missing summary statistics, or data conversions.	Section 2.7. Statistical Analyses
	13c	Describe any methods used to tabulate or visually display results of individual studies and syntheses.	Section 2.7; Tables 2 and 3; Figures 2–4; Supplementary Tables S7–S10
	13d	Describe any methods used to synthesize results and provide a rationale for the choice(s). If meta-analysis was performed, describe the model(s), method(s) to identify the presence and extent of statistical heterogeneity, and software package(s) used.	Section 2.7. Statistical Analyses
	13e	Describe any methods used to explore possible causes of heterogeneity among study results (e.g. subgroup analysis, meta-regression).	Not performed
	13f	Describe any sensitivity analyses conducted to assess robustness of the synthesized results.	Not performed
Reporting bias assessment	14	Describe any methods used to assess risk of bias due to missing results in a synthesis (arising from reporting biases).	Not performed; fewer than 10 studies were available for each meta-analysis (Section 4.9)
Certainty assessment	15	Describe any methods used to assess certainty (or confidence) in the body of evidence for an outcome.	Section 2.7. Statistical Analyses
**RESULTS**			
Study selection	16a	Describe the results of the search and selection process, from the number of records identified in the search to the number of studies included in the review, ideally using a flow diagram.	Section 3.1. Study Selection; Figure 1
	16b	Cite studies that might appear to meet the inclusion criteria, but which were excluded, and explain why they were excluded.	Figure 1
Study characteristics	17	Cite each included study and present its characteristics.	Section 3.2. Characteristics of Included Studies; Tables 2 and 3; Supplementary Tables S7 and S8
Risk of bias in studies	18	Present assessments of risk of bias for each included study.	Section 3.3. Risk of Bias Assessment; Supplementary Figures S1 and S2
Results of individual studies	19	For all outcomes, present, for each study: (a) summary statistics for each group (where appropriate) and (b) an effect estimate and its precision (e.g. confidence/credible interval), ideally using structured tables or plots.	Sections 3.4–3.10; Figures 2–4; Supplementary Tables S9 and S10
Results of syntheses	20a	For each synthesis, briefly summarise the characteristics and risk of bias among contributing studies.	Sections 3.3–3.10; Supplementary Figures S1 and S2
	20b	Present results of all statistical syntheses conducted. If meta-analysis was done, present for each the summary estimate and its precision (e.g. confidence/credible interval) and measures of statistical heterogeneity. If comparing groups, describe the direction of the effect.	Sections 3.4.1, 3.4.2, and 3.5.1; Figures 2–4
	20c	Present results of all investigations of possible causes of heterogeneity among study results.	Not performed
	20d	Present results of all sensitivity analyses conducted to assess the robustness of the synthesized results.	Not performed

Reporting biases	21	Present assessments of risk of bias due to missing results (arising from reporting biases) for each synthesis assessed.	Not performed; fewer than 10 studies were available for each meta-analysis (Section 4.9)
Certainty of evidence	22	Present assessments of certainty (or confidence) in the body of evidence for each outcome assessed.	Supplementary Tables S4–S6
**DISCUSSION**			
Discussion	23a	Provide a general interpretation of the results in the context of other evidence.	Sections 4.1–4.7
	23b	Discuss any limitations of the evidence included in the review.	Section 4.9. Strengths and Limitations
	23c	Discuss any limitations of the review processes used.	Section 4.9. Strengths and Limitations
	23d	Discuss implications of the results for practice, policy, and future research.	Section 4.8. Clinical and Research Implications
**OTHER INFORMATION**			
Registration and protocol	24a	Provide registration information for the review, including register name and registration number, or state that the review was not registered.	Section 2.1. Study Design (PROSPERO, CRD420261452601)
	24b	Indicate where the review protocol can be accessed, or state that a protocol was not prepared.	Section 2.1. Study Design (PROSPERO, CRD420261452601)
	24c	Describe and explain any amendments to information provided at registration or in the protocol.	Section 2.7; the ALT and AST meta-analyses, restricted to placebo-controlled RCTs of silymarin monotherapy, were conducted post hoc
Support	25	Describe sources of financial or non-financial support for the review, and the role of the funders or sponsors in the review.	Funding
Competing interests	26	Declare any competing interests of review authors.	Conflicts of Interest
Availability of data, code and other materials	27	Report which of the following are publicly available and where they can be found: template data collection forms; data extracted from included studies; data used for all analyses; analytic code; any other materials used in the review.	Data Availability Statement; Supplementary Materials

s

Table S2. PRISMA 2020 abstract checklist

Section and Topic	Item #	Checklist item	Reported (Yes/No)
**TITLE**			
Title	1	Identify the report as a systematic review.	Yes
**BACKGROUND**			
Objectives	2	Provide an explicit statement of the main objective(s) or question(s) the review addresses.	Yes
**METHODS**			
Eligibility criteria	3	Specify the inclusion and exclusion criteria for the review.	Yes
Information sources	4	Specify the information sources (e.g. databases, registers) used to identify studies and the date when each was last searched.	Yes
Risk of bias	5	Specify the methods used to assess risk of bias in the included studies.	Yes
Synthesis of results	6	Specify the methods used to present and synthesise results.	Yes
**RESULTS**			
Included studies	7	Give the total number of included studies and participants and summarise relevant characteristics of studies.	Yes
Synthesis of results	8	Present results for main outcomes, preferably indicating the number of included studies and participants for each. If meta-analysis was done, report the summary estimate and confidence/credible interval. If comparing groups, indicate the direction of the effect (i.e. which group is favoured).	Yes
**DISCUSSION**			
Limitations of evidence	9	Provide a brief summary of the limitations of the evidence included in the review (e.g. study risk of bias, inconsistency and imprecision).	Yes
Interpretation	10	Provide a general interpretation of the results and important implications.	Yes
**OTHER**			
Funding	11	Specify the primary source of funding for the review.	No (not reported in the Abstract; see Funding)
Registration	12	Provide the register name and registration number.	No (PROSPERO registration (CRD420261452601) is reported in Methods, not in abstract.)

Table S3. Search strategies for the electronic databases

Database	No.	Search Query
PubMed	#1	"Hepatitis, Chronic"[Mesh]
	#2	"Chronic Hepatitis"[TW] OR "Chronic Active Hepatitis"[TW] OR "Chronic Persistent Hepatitides"[TW] OR "Chronic Persistent Hepatitis"[TW]
	#3	"Liver Cirrhosis"[Mesh]
	#4	"Liver Cirrhosis"[TW] OR "Hepatic Cirrhosis"[TW] OR "cirrhosis"[TW] OR "hepatic fibrosis"[TW]
	#5	"Non-alcoholic Fatty Liver Disease"[Mesh]
	#6	"Non-alcoholic Fatty Liver Disease"[TW] OR "Nonalcoholic Fatty Liver"[TW] OR "Nonalcoholic Fatty Livers"[TW] OR "NAFLD"[TW] OR "Nonalcoholic Fatty Liver Disease"[TW] OR "Nonalcoholic Steatohepatitis"[TW] OR "Nonalcoholic Steatohepatitides"[TW] OR "Metabolic Dysfunction-Associated Steatohepatitis"[TW] OR "non alcoholic fatty liver disease"[TW] OR "non alcoholic hepato-steatosis"[TW] OR "non alcoholic hepatosteatosis"[TW] OR "non alcoholic liver steatosis"[TW] OR "non alcoholic steatotic hepatopathy"[TW] OR "non-alcoholic fatty liver"[TW] OR "non-alcoholic FLD"[TW] OR "non-alcoholic hepatic steatosis"[TW] OR "nonalcoholic FLD"[TW] OR "nonalcoholic hepatic steatosis"[TW] OR "nonalcoholic hepatosteatosis"[TW] OR "nonalcoholic liver steatosis"[TW] OR "Non-alcoholic Steatohepatitis"[TW] OR "NASH"[TW] OR "Metabolic Dysfunction-associated Fatty Liver Disease"[TW] OR "MAFLD"[TW] OR "Metabolic Dysfunction-associated Steatotic Liver Disease"[TW] OR "MASLD"[TW] OR "MASH"[TW]
	#7	"Steatotic Liver Disease"[TW] OR "Steatotic Liver Diseases"[TW] OR "Hepatic Steatosis"[TW]
	#8	#1 OR #2 OR #3 OR #4 OR #5 OR #6 OR #7
	#9	"Silymarin"[Mesh]
	#10	"Silymarin"[TW] OR "Silimarin"[TW] OR "Carsil"[TW] OR "Karsil"[TW] OR "Legalon"[TW] OR "flavobion"[TW] OR "silepar"[TW] OR "silimarine"[TW] OR "silliver"[TW] OR "silymarine"[TW] OR "simepar"[TW] OR "sylimarin"[TW] OR "Silymarins"[TW] OR "silybin"[TW] OR "silybins"[TW] OR "silibinin"[TW] OR "silibinins"[TW] OR "legalons"[TW]
	#11	"Silybum marianum"[Mesh]
	#12	"Silybum marianum"[TW] OR "Milk Thistle"[TW] OR "Milk Thistles"[TW] OR "Carduus marianus"[TW] OR "milk thistle extract"[TW] OR "Silybum marianum extract"[TW]
	#13	#9 OR #10 OR #11 OR #12
	#14	"fibrosis-4 (FIB-4) index"[TW] OR "Fibrosis-4 (FIB-4) index for liver fibrosis"[TW] OR "Fibrosis-4 calculator"[TW] OR "Fibrosis-4 calculator (FIB-4) index"[TW] OR "FIB-4"[TW] OR "FIB4"[TW] OR "Fibrosis-4 Index"[TW] OR "Fibrosis4 Index"[TW]
	#15	"aspartate amino transferase to platelet ratio index"[TW] OR "aspartate aminotransferase/platelet ratio index"[TW] OR "aspartate transaminase (AST) to platelet (PLT) ratio index"[TW] OR "aspartate transaminase (AST) to platelets ratio index"[TW] OR "aspartate transaminase level to platelet count ratio index"[TW] OR "aspartate transaminase to platelet ratio index"[TW] OR "aspartate transaminase to platelets ratio index"[TW] OR "aspartate transaminase to the platelet ratio index"[TW] OR "aspartate transaminase/platelet ratio index"[TW] OR "aspartic aminotransferase to platelet ratio index"[TW] OR "AST to platelet ratio index"[TW] OR "AST to platelets ratio index"[TW] OR "AST/platelet ratio index"[TW] OR "AST/platelets ratio index"[TW] OR "aspartate aminotransferase to platelet ratio index"[TW] OR "APRI"[TW] OR "AST-to-platelet ratio index"[TW]
	#16	"fibrosis index (NAFLD FS)"[TW] OR "NAFLD Fibrosis Scoring (NAFLD-FS or NFS)"[TW] OR "NAFLD-FS"[TW] OR "NAFLD-FS index"[TW] OR "NAFLD-FS score"[TW] OR "non-alcoholic fatty liver disease (NAFLD) fibrosis score"[TW] OR "Non-Alcoholic Fatty Liver Disease Fibrosis Score"[TW] OR "Non-Alcoholic liver fatty liver disease-fibrotic score (NAFLD-FS)"[TW] OR "nonalcoholic fatty liver disease (NAFLD) fibrosis score"[TW] OR "Nonalcoholic Fatty Liver Disease Fibrosis Score"[TW] OR "nonalcoholic fatty liver disease fibrosis score (NAFLD-FS)"[TW] OR "nonalcoholic fatty liver fibrosis score"[TW] OR "NAFLD Fibrosis Score"[TW] OR "NFS"[TW] OR "NAFLD fibrosis score"[TW] OR "NAFLD fibrosis scores"[TW]
	#17	"Enhanced liver fibrosis"[TW] OR "ELF score"[TW] OR "ELF scores"[TW] OR "ELF test"[TW] OR "ELF tests"[TW]
	#18	"Alanine Transaminase"[Mesh]
	#19	"Alanine Transaminase"[TW] OR "Alanine Aminotransferase"[TW] OR "Glutamic-Alanine Transaminase"[TW] OR "Glutamic-Pyruvic Transaminase"[TW] OR "Alanine-2-Oxoglutarate Aminotransferase"[TW] OR "SGPT"[TW] OR "alanin aminotransferase"[TW] OR "alanine 2 oxoglutarate aminotransferase"[TW] OR "alanine 2 oxoisovalerate aminotransferase"[TW] OR "alanine alpha ketoglutarate transaminase"[TW] OR "alanine alpha oxoglutarate transaminase"[TW] OR "alanine amino transferase"[TW]

		OR "alanine transpeptidase"[TW] OR "alanine:2 oxoglutarate aminotransferase"[TW] OR "e.c. 2.6.1.2"[TW] OR "glutamate alanine transaminase"[TW] OR "glutamate pyruvate aminotransferase"[TW] OR "glutamate pyruvate transaminase"[TW] OR "glutamate pyruvatetransaminase"[TW] OR "glutamic alanine aminotransferase"[TW] OR "glutamic pyruvate transaminase"[TW] OR "glutamic pyruvic aminotransferase"[TW] OR "glutamic pyruvic transaminase"[TW] OR "glutamopyruvic transaminase"[TW] OR "GPT"[TW] OR "l alanine 2 oxoglutarate aminotransferase"[TW] OR "l alanine:2 oxoglutarate aminotransferase"[TW] OR "ALT"[TW] OR "alanine transaminases"[TW] OR "alanine aminotransferases"[TW]
	#20	"Aspartate Aminotransferases"[Mesh]
	#21	"Aspartate Aminotransferases"[TW] OR "Glutamic-Oxaloacetic Transaminase"[TW] OR "L-Aspartate-2-Oxoglutarate Aminotransferase"[TW] OR "Glutamate-Aspartate Transaminase"[TW] OR "Aspartate Aminotransferase"[TW] OR "Aspartate Apoaminotransferase"[TW] OR "Aspartate Transaminase"[TW] OR "SGOT"[TW] OR "Serum Glutamic-Oxaloacetic Transaminase"[TW] OR "1 aspartate 2 oxoglutarate aminotransferase"[TW] OR "1 aspartate:2 oxoglutarate aminotransferase"[TW] OR "aminotransferase, aspartate"[TW] OR "aspartate amino transferase"[TW] OR "aspartic aminotransferase"[TW] OR "aspartic transaminase"[TW] OR "e.c. 2.6.1.1"[TW] OR "glutamate aspartate transaminase"[TW] OR "glutamate oxalacetate transaminase"[TW] OR "glutamate oxalacetic transaminase"[TW] OR "glutamate oxalate transaminase"[TW] OR "glutamate oxalic transaminase"[TW] OR "glutamate oxaloacetate aminotransferase"[TW] OR "glutamate oxaloacetate transaminase"[TW] OR "glutamate oxaloacetic acid transaminase"[TW] OR "glutamatoxalacetate transaminase"[TW] OR "glutamic aspartic aminotransferase"[TW] OR "glutamic aspartic transaminase"[TW] OR "glutamic oxal acetatic transaminase"[TW] OR "glutamic oxalacetic acid transaminase"[TW] OR "glutamic oxalacetic transaminase"[TW] OR "glutamic oxalacetic transferase"[TW] OR "glutamic oxalic transaminase"[TW] OR "glutamic oxaloacetic acid transaminase"[TW] OR "glutamic oxaloacetic aminotransferase"[TW] OR "glutamic oxaloacetic transaminase"[TW] OR "glutamine oxaloacetic transaminase"[TW] OR "glutaminic oxalacetic transaminase"[TW] OR "GOT"[TW] OR "l asparate 2 oxoglutarate transaminase"[TW] OR "l aspartate 2 oxoglutarate aminotransferase"[TW] OR "l aspartate aminotransferase"[TW] OR "l aspartate:2 oxoglutarate aminotransferase"[TW] OR "l aspartate:2 oxoglutarate transaminase"[TW] OR "l aspatate:2 oxoglutarate aminotransferase"[TW] OR "levo aspartate aminotransferase"[TW] OR "transaminase a"[TW] OR "aspartate aminotransferase"[TW] OR "AST"[TW] OR "aspartate transaminases"[TW]
	#22	"gamma-Glutamyltransferase"[Mesh]
	#23	"gamma-Glutamyltransferase"[TW] OR "gamma-Glutamyl Transpeptidase"[TW] OR "gammaglutamyltransferase"[TW] OR "GGTP"[TW] OR "Glutamyl Transpeptidase"[TW] OR "(5 glutamyl) peptide:amino acid 5 glutamyltransferase"[TW] OR "4 glutamyl transferase"[TW] OR "alpha glutamyltranspeptidase"[TW] OR "e.c. 2.3.2.2"[TW] OR "gamma glutamyl transferase"[TW] OR "gamma glutamyl transpeptidase"[TW] OR "gamma glutamyltranspeptidase"[TW] OR "gamma gt"[TW] OR "gamma-glutamyltransferase"[TW] OR "gammaglutamyl transpeptidase"[TW] OR "glutamate transpeptidase"[TW] OR "glutamic transferase"[TW] OR "glutamyl transferase"[TW] OR "glutamyl transpeptidase"[TW] OR "glutamyltransferase"[TW] OR "glutamyltranspeptidase"[TW] OR "levo glutamyltranspeptidase"[TW] OR "transpeptidase"[TW] OR "GGT"[TW] OR "gamma-glutamyltransferases"[TW] OR "gamma-glutamyl transpeptidase"[TW] OR "gamma-glutamyl transpeptidases"[TW]
	#24	"liver cell enzyme"[TW] OR "liver enzyme"[TW] OR "liver enzymes"[TW] OR "hepatic enzyme"[TW] OR "hepatic enzymes"[TW]
	#25	"Transaminases"[Mesh]
	#26	"Aminotransferase"[TW] OR "Aminotransferases"[TW] OR "alpha oxoglutarate transferase"[TW] OR "amino transferase"[TW] OR "d54.680"[TW] OR "transaminase"[TW] OR "transaminases"[TW]
	#27	"Liver Function Tests"[Mesh]
	#28	"Liver Function Tests"[TW] OR "Liver Function Test"[TW] OR "hepatic function test"[TW] OR "liverfunction test"[TW] OR "LFT"[TW] OR "LFTs"[TW]
	#29	"liver stiffness"[TW] OR "hepatic stiffness"[TW] OR "LSM"[TW]
	#30	"transient elastography"[TW] OR "transient elastography technique"[TW]
	#31	"elastograph"[TW] OR "hepatic ultrasound elastography system"[TW] OR "hepatic ultrasound elastography system applicator"[TW] OR "ultrasound elastography system"[TW] OR "ultrasound elastography system applicator"[TW] OR "shear wave elastography"[TW] OR "SWE"[TW] OR "magnetic resonance elastography"[TW] OR "MRE"[TW] OR "fatty liver index"[TW] OR "FLI"[TW] OR "fatty liver score"[TW] OR "FLS"[TW] OR "fibrosis stage"[TW] OR "histological fibrosis"[TW] OR (histolog*[TW] AND fibros*[TW]) OR (biops*[TW] AND fibros*[TW])
	#32	#14 OR #15 OR #16 OR #17 OR #18 OR #19 OR #20 OR #21 OR #22 OR #23 OR #24 OR #25 OR #26 OR #27 OR #28 OR #29 OR #30 OR #31

	#33	#8 AND #13 AND #32

Database	No.	Search Query
EMBASE	#1	"chronic hepatitis"/exp
	#2	"Chronic Hepatitis":ti,ab,kw OR "Chronic Active Hepatitis":ti,ab,kw OR "Chronic Persistent Hepatitides":ti,ab,kw OR "Chronic Persistent Hepatitis":ti,ab,kw
	#3	"liver cirrhosis"/exp
	#4	"Liver Cirrhosis":ti,ab,kw OR "Hepatic Cirrhosis":ti,ab,kw OR "cirrhosis":ti,ab,kw OR "hepatic fibrosis":ti,ab,kw
	#5	"nonalcoholic fatty liver"/exp
	#6	"Non-alcoholic Fatty Liver Disease":ti,ab,kw OR "Nonalcoholic Fatty Liver":ti,ab,kw OR "Nonalcoholic Fatty Livers":ti,ab,kw OR "NAFLD":ti,ab,kw OR "Nonalcoholic Fatty Liver Disease":ti,ab,kw OR "Nonalcoholic Steatohepatitis":ti,ab,kw OR "Nonalcoholic Steatohepatitides":ti,ab,kw OR "Metabolic Dysfunction-Associated Steatohepatitis":ti,ab,kw OR "non alcoholic fatty liver disease":ti,ab,kw OR "non alcoholic hepato-steatosis":ti,ab,kw OR "non alcoholic hepatosteatosis":ti,ab,kw OR "non alcoholic liver steatosis":ti,ab,kw OR "non alcoholic steatotic hepatopathy":ti,ab,kw OR "non-alcoholic fatty liver":ti,ab,kw OR "non-alcoholic FLD":ti,ab,kw OR "non-alcoholic hepatic steatosis":ti,ab,kw OR "nonalcoholic FLD":ti,ab,kw OR "nonalcoholic hepatic steatosis":ti,ab,kw OR "nonalcoholic hepatosteatosis":ti,ab,kw OR "nonalcoholic liver steatosis":ti,ab,kw OR "Non-alcoholic Steatohepatitis":ti,ab,kw OR "NASH":ti,ab,kw OR "Metabolic Dysfunction-associated Fatty Liver Disease":ti,ab,kw OR "MAFLD":ti,ab,kw OR "Metabolic Dysfunction-associated Steatotic Liver Disease":ti,ab,kw OR "MASLD":ti,ab,kw OR "MASH":ti,ab,kw
	#7	"Steatotic Liver Disease":ti,ab,kw OR "Steatotic Liver Diseases":ti,ab,kw OR "Hepatic Steatosis":ti,ab,kw
	#8	#1 OR #2 OR #3 OR #4 OR #5 OR #6 OR #7
	#9	"silymarin"/exp
	#10	"Silymarin":ti,ab,kw OR "Silimarin":ti,ab,kw OR "Carsil":ti,ab,kw OR "Karsil":ti,ab,kw OR "Legalon":ti,ab,kw OR "flavobion":ti,ab,kw OR "silepar":ti,ab,kw OR "silimarine":ti,ab,kw OR "silliver":ti,ab,kw OR "silymarine":ti,ab,kw OR "simepar":ti,ab,kw OR "sylimarin":ti,ab,kw OR "Silymarins":ti,ab,kw OR "silybin":ti,ab,kw OR "silybins":ti,ab,kw OR "silibinin":ti,ab,kw OR "silibinins":ti,ab,kw OR "legalons":ti,ab,kw
	#11	"Silybum marianum"/exp
	#12	"Silybum marianum":ti,ab,kw OR "Milk Thistle":ti,ab,kw OR "Milk Thistles":ti,ab,kw OR "Carduus marianus":ti,ab,kw OR "milk thistle extract":ti,ab,kw OR "Silybum marianum extract":ti,ab,kw
	#13	#9 OR #10 OR #11 OR #12
	#14	"Fibrosis-4 Index"/exp OR "fibrosis-4 (FIB-4) index":ti,ab,kw OR "Fibrosis-4 (FIB-4) index for liver fibrosis":ti,ab,kw OR "Fibrosis-4 calculator":ti,ab,kw OR "Fibrosis-4 calculator (FIB-4) index":ti,ab,kw OR "FIB-4":ti,ab,kw OR "FIB4":ti,ab,kw OR "Fibrosis-4 Index":ti,ab,kw OR "Fibrosis4 Index":ti,ab,kw
	#15	"aspartate aminotransferase to platelet ratio index"/exp OR "aspartate amino transferase to platelet ratio index":ti,ab,kw OR "aspartate aminotransferase/platelet ratio index":ti,ab,kw OR "aspartate transaminase (AST) to platelet (PLT) ratio index":ti,ab,kw OR "aspartate transaminase (AST) to platelets ratio index":ti,ab,kw OR "aspartate transaminase level to platelet count ratio index":ti,ab,kw OR "aspartate transaminase to platelet ratio index":ti,ab,kw OR "aspartate transaminase to platelets ratio index":ti,ab,kw OR "aspartate transaminase to the platelet ratio index":ti,ab,kw OR "aspartate transaminase/platelet ratio index":ti,ab,kw OR "aspartic aminotransferase to platelet ratio index":ti,ab,kw OR "AST to platelet ratio index":ti,ab,kw OR "AST to platelets ratio index":ti,ab,kw OR "AST/platelet ratio index":ti,ab,kw OR "AST/platelets ratio index":ti,ab,kw OR "aspartate aminotransferase to platelet ratio index":ti,ab,kw OR "APRI":ti,ab,kw OR "AST-to-platelet ratio index":ti,ab,kw
	#16	"NAFLD Fibrosis Score"/exp OR "fibrosis index (NAFLD FS)":ti,ab,kw OR "NAFLD Fibrosis Scoring (NAFLD-FS or NFS)":ti,ab,kw OR "NAFLD-FS":ti,ab,kw OR "NAFLD-FS index":ti,ab,kw OR "NAFLD-FS score":ti,ab,kw OR "non-alcoholic fatty liver disease (NAFLD) fibrosis score":ti,ab,kw OR "Non-Alcoholic Fatty Liver Disease Fibrosis Score":ti,ab,kw OR "Non-Alcoholic liver fatty liver disease-fibrotic score (NAFLD-FS)":ti,ab,kw OR "nonalcoholic fatty liver disease (NAFLD) fibrosis score":ti,ab,kw OR "Nonalcoholic Fatty Liver Disease Fibrosis Score":ti,ab,kw OR "nonalcoholic fatty liver disease fibrosis score (NAFLD-FS)":ti,ab,kw OR "nonalcoholic fatty liver fibrosis score":ti,ab,kw OR "NAFLD Fibrosis Score":ti,ab,kw OR "NFS":ti,ab,kw OR "NAFLD fibrosis score":ti,ab,kw OR "NAFLD fibrosis scores":ti,ab,kw
	#17	"Enhanced liver fibrosis":ti,ab,kw OR "ELF score":ti,ab,kw OR "ELF scores":ti,ab,kw OR "ELF test":ti,ab,kw OR "ELF tests":ti,ab,kw
	#18	"alanine aminotransferase"/exp

	#19	"Alanine Transaminase":ti,ab,kw OR "Alanine Aminotransferase":ti,ab,kw OR "Glutamic-Alanine Transaminase":ti,ab,kw OR "Glutamic-Pyruvic Transaminase":ti,ab,kw OR "Alanine-2-Oxoglutarate Aminotransferase":ti,ab,kw OR "SGPT":ti,ab,kw OR "alanin aminotransferase":ti,ab,kw OR "alanine 2 oxoglutarate aminotransferase":ti,ab,kw OR "alanine 2 oxoisovalerate aminotransferase":ti,ab,kw OR "alanine alpha ketoglutarate transaminase":ti,ab,kw OR "alanine alpha oxoglutarate transaminase":ti,ab,kw OR "alanine amino transferase":ti,ab,kw OR "alanine transpeptidase":ti,ab,kw OR "alanine:2 oxoglutarate aminotransferase":ti,ab,kw OR "e.c. 2.6.1.2":ti,ab,kw OR "glutamate alanine transaminase":ti,ab,kw OR "glutamate pyruvate aminotransferase":ti,ab,kw OR "glutamate pyruvate transaminase":ti,ab,kw OR "glutamate pyruvatetransaminase":ti,ab,kw OR "glutamic alanine aminotransferase":ti,ab,kw OR "glutamic pyruvate transaminase":ti,ab,kw OR "glutamic pyruvic aminotransferase":ti,ab,kw OR "glutamic pyruvic transaminase":ti,ab,kw OR "glutamopyruvic transaminase":ti,ab,kw OR "GPT":ti,ab,kw OR "l alanine 2 oxoglutarate aminotransferase":ti,ab,kw OR "l alanine:2 oxoglutarate aminotransferase":ti,ab,kw OR "ALT":ti,ab,kw OR "alanine transaminases":ti,ab,kw OR "alanine aminotransferases":ti,ab,kw
	#20	"aspartate aminotransferase"/exp
	#21	"Aspartate Aminotransferases":ti,ab,kw OR "Glutamic-Oxaloacetic Transaminase":ti,ab,kw OR "L-Aspartate-2-Oxoglutarate Aminotransferase":ti,ab,kw OR "Glutamate-Aspartate Transaminase":ti,ab,kw OR "Aspartate Aminotransferase":ti,ab,kw OR "Aspartate Apoaminotransferase":ti,ab,kw OR "Aspartate Transaminase":ti,ab,kw OR "SGOT":ti,ab,kw OR "Serum Glutamic-Oxaloacetic Transaminase":ti,ab,kw OR "l aspartate 2 oxoglutarate aminotransferase":ti,ab,kw OR "l aspartate:2 oxoglutarate aminotransferase":ti,ab,kw OR "aminotransferase, aspartate":ti,ab,kw OR "aspartate amino transferase":ti,ab,kw OR "aspartic aminotransferase":ti,ab,kw OR "aspartic transaminase":ti,ab,kw OR "e.c. 2.6.1.1":ti,ab,kw OR "glutamate aspartate transaminase":ti,ab,kw OR "glutamate oxalacetate transaminase":ti,ab,kw OR "glutamate oxalacetic transaminase":ti,ab,kw OR "glutamate oxalate transaminase":ti,ab,kw OR "glutamate oxalic transaminase":ti,ab,kw OR "glutamate oxaloacetate aminotransferase":ti,ab,kw OR "glutamate oxaloacetate transaminase":ti,ab,kw OR "glutamate oxaloacetic acid transaminase":ti,ab,kw OR "glutamatoxalacetate transaminase":ti,ab,kw OR "glutamic aspartic aminotransferase":ti,ab,kw OR "glutamic aspartic transaminase":ti,ab,kw OR "glutamic oxal acetatic transaminase":ti,ab,kw OR "glutamic oxalacetic acid transaminase":ti,ab,kw OR "glutamic oxalacetic transaminase":ti,ab,kw OR "glutamic oxalacetic transferase":ti,ab,kw OR "glutamic oxalic transaminase":ti,ab,kw OR "glutamic oxaloacetic acid transaminase":ti,ab,kw OR "glutamic oxaloacetic aminotransferase":ti,ab,kw OR "glutamic oxaloacetic transaminase":ti,ab,kw OR "glutamine oxaloacetic transaminase":ti,ab,kw OR "glutaminic oxalacetic transaminase":ti,ab,kw OR "GOT":ti,ab,kw OR "l asparate 2 oxoglutarate transaminase":ti,ab,kw OR "l aspartate 2 oxoglutarate aminotransferase":ti,ab,kw OR "l aspartate aminotransferase":ti,ab,kw OR "l aspartate:2 oxoglutarate aminotransferase":ti,ab,kw OR "l aspartate:2 oxoglutarate transaminase":ti,ab,kw OR "l aspatate:2 oxoglutarate aminotransferase":ti,ab,kw OR "levo aspartate aminotransferase":ti,ab,kw OR "transaminase a":ti,ab,kw OR "aspartate aminotransferase":ti,ab,kw OR "AST":ti,ab,kw OR "aspartate transaminases":ti,ab,kw
	#22	"gamma glutamyltransferase"/exp
	#23	"gamma-Glutamyltransferase":ti,ab,kw OR "gamma-Glutamyl Transpeptidase":ti,ab,kw OR "gammaglutamyltransferase":ti,ab,kw OR "GGTP":ti,ab,kw OR "Glutamyl Transpeptidase":ti,ab,kw OR "(5 glutamyl) peptide:amino acid 5 glutamyltransferase":ti,ab,kw OR "4 glutamyl transferase":ti,ab,kw OR "alpha glutamyltranspeptidase":ti,ab,kw OR "e.c. 2.3.2.2":ti,ab,kw OR "gamma glutamyl transferase":ti,ab,kw OR "gamma glutamyl transpeptidase":ti,ab,kw OR "gamma glutamyltranspeptidase":ti,ab,kw OR "gamma gt":ti,ab,kw OR "gamma-glutamyltransferase":ti,ab,kw OR "gammaglutamyl transpeptidase":ti,ab,kw OR "glutamate transpeptidase":ti,ab,kw OR "glutamic transferase":ti,ab,kw OR "glutamyl transferase":ti,ab,kw OR "glutamyl transpeptidase":ti,ab,kw OR "glutamyltransferase":ti,ab,kw OR "glutamyltranspeptidase":ti,ab,kw OR "levo glutamyltranspeptidase":ti,ab,kw OR "transpeptidase":ti,ab,kw OR "GGT":ti,ab,kw OR "gamma-glutamyltransferases":ti,ab,kw OR "gamma-glutamyl transpeptidase":ti,ab,kw OR "gamma-glutamyl transpeptidases":ti,ab,kw
	#24	"liver enzyme"/exp OR "liver cell enzyme":ti,ab,kw OR "liver enzyme":ti,ab,kw OR "liver enzymes":ti,ab,kw OR "hepatic enzyme":ti,ab,kw OR "hepatic enzymes":ti,ab,kw
	#25	"aminotransferase"/exp
	#26	"Aminotransferase":ti,ab,kw OR "Aminotransferases":ti,ab,kw OR "alpha oxoglutarate transferase":ti,ab,kw OR "amino transferase":ti,ab,kw OR "d54.680":ti,ab,kw OR "transaminase":ti,ab,kw OR "transaminases":ti,ab,kw
	#27	"liver function test"/exp
	#28	"liver stiffness"/exp OR "Liver Function Tests":ti,ab,kw OR "Liver Function Test":ti,ab,kw OR "hepatic function test":ti,ab,kw OR "liverfunction test":ti,ab,kw OR "LFT":ti,ab,kw OR "LFTs":ti,ab,kw
	#29	"liver stiffness":ti,ab,kw OR "hepatic stiffness":ti,ab,kw OR "LSM":ti,ab,kw

	#30	"transient elastography"/exp OR "transient elastography":ti,ab,kw OR "transient elastography technique":ti,ab,kw
	#31	"elastograph"/exp OR "elastograph":ti,ab,kw OR "hepatic ultrasound elastography system":ti,ab,kw OR "hepatic ultrasound elastography system applicator":ti,ab,kw OR "ultrasound elastography system":ti,ab,kw OR "ultrasound elastography system applicator":ti,ab,kw OR "shear wave elastography":ti,ab,kw OR "SWE":ti,ab,kw OR "magnetic resonance elastography":ti,ab,kw OR "MRE":ti,ab,kw OR "fatty liver index":ti,ab,kw OR "FLI":ti,ab,kw OR "fatty liver score":ti,ab,kw OR "FLS":ti,ab,kw OR "fibrosis stage":ti,ab,kw OR "histological fibrosis":ti,ab,kw OR (histolog*:ti,ab,kw AND fibros*:ti,ab,kw) OR (biops*:ti,ab,kw AND fibros*:ti,ab,kw)
	#32	#14 OR #15 OR #16 OR #17 OR #18 OR #19 OR #20 OR #21 OR #22 OR #23 OR #24 OR #25 OR #26 OR #27 OR #28 OR #29 OR #30 OR #31
	#33	#8 AND #13 AND #32

Database	No.	Search Query
Cochrane Library	#1	[mh "Hepatitis, Chronic"]
	#2	"Chronic Hepatitis":ti,ab,kw OR "Chronic Active Hepatitis":ti,ab,kw OR "Chronic Persistent Hepatitides":ti,ab,kw OR "Chronic Persistent Hepatitis":ti,ab,kw
	#3	[mh "Liver Cirrhosis"]
	#4	"Liver Cirrhosis":ti,ab,kw OR "Hepatic Cirrhosis":ti,ab,kw OR "cirrhosis":ti,ab,kw OR "hepatic fibrosis":ti,ab,kw
	#5	[mh "Non-alcoholic Fatty Liver Disease"]
	#6	"Non-alcoholic Fatty Liver Disease":ti,ab,kw OR "Nonalcoholic Fatty Liver":ti,ab,kw OR "Nonalcoholic Fatty Livers":ti,ab,kw OR "NAFLD":ti,ab,kw OR "Nonalcoholic Fatty Liver Disease":ti,ab,kw OR "Nonalcoholic Steatohepatitis":ti,ab,kw OR "Nonalcoholic Steatohepatitides":ti,ab,kw OR "Metabolic Dysfunction-Associated Steatohepatitis":ti,ab,kw OR "non alcoholic fatty liver disease":ti,ab,kw OR "non alcoholic hepato-steatosis":ti,ab,kw OR "non alcoholic hepatosteatosis":ti,ab,kw OR "non alcoholic liver steatosis":ti,ab,kw OR "non alcoholic steatotic hepatopathy":ti,ab,kw OR "non-alcoholic fatty liver":ti,ab,kw OR "non-alcoholic FLD":ti,ab,kw OR "non-alcoholic hepatic steatosis":ti,ab,kw OR "nonalcoholic FLD":ti,ab,kw OR "nonalcoholic hepatic steatosis":ti,ab,kw OR "nonalcoholic hepatosteatosis":ti,ab,kw OR "nonalcoholic liver steatosis":ti,ab,kw OR "Non-alcoholic Steatohepatitis":ti,ab,kw OR "NASH":ti,ab,kw OR "Metabolic Dysfunction-associated Fatty Liver Disease":ti,ab,kw OR "MAFLD":ti,ab,kw OR "Metabolic Dysfunction-associated Steatotic Liver Disease":ti,ab,kw OR "MASLD":ti,ab,kw OR "MASH":ti,ab,kw
	#7	"Steatotic Liver Disease":ti,ab,kw OR "Steatotic Liver Diseases":ti,ab,kw OR "Hepatic Steatosis":ti,ab,kw
	#8	#1 OR #2 OR #3 OR #4 OR #5 OR #6 OR #7
	#9	[mh "Silymarin"]
	#10	"Silymarin":ti,ab,kw OR "Silimarin":ti,ab,kw OR "Carsil":ti,ab,kw OR "Karsil":ti,ab,kw OR "Legalon":ti,ab,kw OR "flavobion":ti,ab,kw OR "silepar":ti,ab,kw OR "silimarine":ti,ab,kw OR "silliver":ti,ab,kw OR "silymarine":ti,ab,kw OR "simepar":ti,ab,kw OR "sylimarin":ti,ab,kw OR "Silymarins":ti,ab,kw OR "silybin":ti,ab,kw OR "silybins":ti,ab,kw OR "silibinin":ti,ab,kw OR "silibinins":ti,ab,kw OR "legalons":ti,ab,kw
	#11	[mh "Silybum marianum"]
	#12	"Silybum marianum":ti,ab,kw OR "Milk Thistle":ti,ab,kw OR "Milk Thistles":ti,ab,kw OR "Carduus marianus":ti,ab,kw OR "milk thistle extract":ti,ab,kw OR "Silybum marianum extract":ti,ab,kw
	#13	#9 OR #10 OR #11 OR #12
	#14	"fibrosis-4 (FIB-4) index":ti,ab,kw OR "Fibrosis-4 (FIB-4) index for liver fibrosis":ti,ab,kw OR "Fibrosis-4 calculator":ti,ab,kw OR "Fibrosis-4 calculator (FIB-4) index":ti,ab,kw OR "FIB-4":ti,ab,kw OR "FIB4":ti,ab,kw OR "Fibrosis-4 Index":ti,ab,kw OR "Fibrosis4 Index":ti,ab,kw
	#15	"aspartate amino transferase to platelet ratio index":ti,ab,kw OR "aspartate aminotransferase/platelet ratio index":ti,ab,kw OR "aspartate transaminase (AST) to platelet (PLT) ratio index":ti,ab,kw OR "aspartate transaminase (AST) to platelets ratio index":ti,ab,kw OR "aspartate transaminase level to platelet count ratio index":ti,ab,kw OR "aspartate transaminase to platelet ratio index":ti,ab,kw OR "aspartate transaminase to platelets ratio index":ti,ab,kw OR "aspartate transaminase to the platelet ratio index":ti,ab,kw OR "aspartate transaminase/platelet ratio index":ti,ab,kw OR "aspartic aminotransferase to platelet ratio index":ti,ab,kw OR "AST to platelet ratio index":ti,ab,kw OR "AST to platelets ratio index":ti,ab,kw OR "AST/platelet ratio index":ti,ab,kw OR "AST/platelets ratio index":ti,ab,kw OR "aspartate aminotransferase to platelet ratio index":ti,ab,kw OR "APRI":ti,ab,kw OR "AST-to-platelet ratio index":ti,ab,kw
	#16	"fibrosis index (NAFLD FS)":ti,ab,kw OR "NAFLD Fibrosis Scoring (NAFLD-FS or NFS)":ti,ab,kw OR "NAFLD-FS":ti,ab,kw OR "NAFLD-FS index":ti,ab,kw OR "NAFLD-FS score":ti,ab,kw OR "non-alcoholic fatty liver disease (NAFLD) fibrosis score":ti,ab,kw OR "Non-Alcoholic Fatty Liver Disease Fibrosis

		Score":ti,ab,kw OR "Non-Alcoholic liver fatty liver disease-fibrotic score (NAFLD-FS)":ti,ab,kw OR "nonalcoholic fatty liver disease (NAFLD) fibrosis score":ti,ab,kw OR "Nonalcoholic Fatty Liver Disease Fibrosis Score":ti,ab,kw OR "nonalcoholic fatty liver disease fibrosis score (NAFLD-FS)":ti,ab,kw OR "nonalcoholic fatty liver fibrosis score":ti,ab,kw OR "NAFLD Fibrosis Score":ti,ab,kw OR "NFS":ti,ab,kw OR "NAFLD fibrosis score":ti,ab,kw OR "NAFLD fibrosis scores":ti,ab,kw
	#17	"Enhanced liver fibrosis":ti,ab,kw OR "ELF score":ti,ab,kw OR "ELF scores":ti,ab,kw OR "ELF test":ti,ab,kw OR "ELF tests":ti,ab,kw
	#18	[mh "Alanine Transaminase"]
	#19	"Alanine Transaminase":ti,ab,kw OR "Alanine Aminotransferase":ti,ab,kw OR "Glutamic-Alanine Transaminase":ti,ab,kw OR "Glutamic-Pyruvic Transaminase":ti,ab,kw OR "Alanine-2-Oxoglutarate Aminotransferase":ti,ab,kw OR "SGPT":ti,ab,kw OR "alanin aminotransferase":ti,ab,kw OR "alanine 2 oxoglutarate aminotransferase":ti,ab,kw OR "alanine 2 oxoisovalerate aminotransferase":ti,ab,kw OR "alanine alpha ketoglutarate transaminase":ti,ab,kw OR "alanine alpha oxoglutarate transaminase":ti,ab,kw OR "alanine amino transferase":ti,ab,kw OR "alanine transpeptidase":ti,ab,kw OR "alanine:2 oxoglutarate aminotransferase":ti,ab,kw OR "e.c. 2.6.1.2":ti,ab,kw OR "glutamate alanine transaminase":ti,ab,kw OR "glutamate pyruvate aminotransferase":ti,ab,kw OR "glutamate pyruvate transaminase":ti,ab,kw OR "glutamate pyruvatetransaminase":ti,ab,kw OR "glutamic alanine aminotransferase":ti,ab,kw OR "glutamic pyruvate transaminase":ti,ab,kw OR "glutamic pyruvic aminotransferase":ti,ab,kw OR "glutamic pyruvic transaminase":ti,ab,kw OR "glutamopyruvic transaminase":ti,ab,kw OR "GPT":ti,ab,kw OR "l alanine 2 oxoglutarate aminotransferase":ti,ab,kw OR "l alanine:2 oxoglutarate aminotransferase":ti,ab,kw OR "ALT":ti,ab,kw OR "alanine transaminases":ti,ab,kw OR "alanine aminotransferases":ti,ab,kw
	#20	[mh "Aspartate Aminotransferases"]
	#21	"Aspartate Aminotransferases":ti,ab,kw OR "Glutamic-Oxaloacetic Transaminase":ti,ab,kw OR "L-Aspartate-2-Oxoglutarate Aminotransferase":ti,ab,kw OR "Glutamate-Aspartate Transaminase":ti,ab,kw OR "Aspartate Aminotransferase":ti,ab,kw OR "Aspartate Apoaminotransferase":ti,ab,kw OR "Aspartate Transaminase":ti,ab,kw OR "SGOT":ti,ab,kw OR "Serum Glutamic-Oxaloacetic Transaminase":ti,ab,kw OR "l aspartate 2 oxoglutarate aminotransferase":ti,ab,kw OR "l aspartate:2 oxoglutarate aminotransferase":ti,ab,kw OR "aminotransferase, aspartate":ti,ab,kw OR "aspartate amino transferase":ti,ab,kw OR "aspartic aminotransferase":ti,ab,kw OR "aspartic transaminase":ti,ab,kw OR "e.c. 2.6.1.1":ti,ab,kw OR "glutamate aspartate transaminase":ti,ab,kw OR "glutamate oxalacetate transaminase":ti,ab,kw OR "glutamate oxalacetic transaminase":ti,ab,kw OR "glutamate oxalate transaminase":ti,ab,kw OR "glutamate oxalic transaminase":ti,ab,kw OR "glutamate oxaloacetate aminotransferase":ti,ab,kw OR "glutamate oxaloacetate transaminase":ti,ab,kw OR "glutamate oxaloacetic acid transaminase":ti,ab,kw OR "glutamatoxalacetate transaminase":ti,ab,kw OR "glutamic aspartic aminotransferase":ti,ab,kw OR "glutamic aspartic transaminase":ti,ab,kw OR "glutamic oxal acetatic transaminase":ti,ab,kw OR "glutamic oxalacetic acid transaminase":ti,ab,kw OR "glutamic oxalacetic transaminase":ti,ab,kw OR "glutamic oxalacetic transferase":ti,ab,kw OR "glutamic oxalic transaminase":ti,ab,kw OR "glutamic oxaloacetic acid transaminase":ti,ab,kw OR "glutamic oxaloacetic aminotransferase":ti,ab,kw OR "glutamic oxaloacetic transaminase":ti,ab,kw OR "glutamine oxaloacetic transaminase":ti,ab,kw OR "glutaminic oxalacetic transaminase":ti,ab,kw OR "GOT":ti,ab,kw OR "l asparate 2 oxoglutarate transaminase":ti,ab,kw OR "l aspartate 2 oxoglutarate aminotransferase":ti,ab,kw OR "l aspartate aminotransferase":ti,ab,kw OR "l aspartate:2 oxoglutarate aminotransferase":ti,ab,kw OR "l aspartate:2 oxoglutarate transaminase":ti,ab,kw OR "l aspatate:2 oxoglutarate aminotransferase":ti,ab,kw OR "levo aspartate aminotransferase":ti,ab,kw OR "transaminase a":ti,ab,kw OR "aspartate aminotransferase":ti,ab,kw OR "AST":ti,ab,kw OR "aspartate transaminases":ti,ab,kw
	#22	[mh "gamma-Glutamyltransferase"]
	#23	"gamma-Glutamyltransferase":ti,ab,kw OR "gamma-Glutamyl Transpeptidase":ti,ab,kw OR "gammaglutamyltransferase":ti,ab,kw OR "GGTP":ti,ab,kw OR "Glutamyl Transpeptidase":ti,ab,kw OR "(5 glutamyl) peptide:amino acid 5 glutamyltransferase":ti,ab,kw OR "4 glutamyl transferase":ti,ab,kw OR "alpha glutamyltranspeptidase":ti,ab,kw OR "e.c. 2.3.2.2":ti,ab,kw OR "gamma glutamyl transferase":ti,ab,kw OR "gamma glutamyl transpeptidase":ti,ab,kw OR "gamma glutamyltranspeptidase":ti,ab,kw OR "gamma gt":ti,ab,kw OR "gamma-glutamyltransferase":ti,ab,kw OR "gammaglutamyl transpeptidase":ti,ab,kw OR "glutamate transpeptidase":ti,ab,kw OR "glutamic transferase":ti,ab,kw OR "glutamyl transferase":ti,ab,kw OR "glutamyl transpeptidase":ti,ab,kw OR "glutamyltransferase":ti,ab,kw OR "glutamyltranspeptidase":ti,ab,kw OR "levo glutamyltranspeptidase":ti,ab,kw OR "transpeptidase":ti,ab,kw OR "GGT":ti,ab,kw OR "gamma-glutamyltransferases":ti,ab,kw OR "gamma-glutamyl transpeptidase":ti,ab,kw OR "gamma-glutamyl transpeptidases":ti,ab,kw
	#24	"liver cell enzyme":ti,ab,kw OR "liver enzyme":ti,ab,kw OR "liver enzymes":ti,ab,kw OR "hepatic enzyme":ti,ab,kw OR "hepatic enzymes":ti,ab,kw
	#25	[mh "Transaminases"]

	#26	"Aminotransferase":ti,ab,kw OR "Aminotransferases":ti,ab,kw OR "alpha oxoglutarate transferase":ti,ab,kw OR "amino transferase":ti,ab,kw OR "d54.680":ti,ab,kw OR "transaminase":ti,ab,kw OR "transaminases":ti,ab,kw
	#27	[mh "Liver Function Tests"]
	#28	"Liver Function Tests":ti,ab,kw OR "Liver Function Test":ti,ab,kw OR "hepatic function test":ti,ab,kw OR "liverfunction test":ti,ab,kw OR "LFT":ti,ab,kw OR "LFTs":ti,ab,kw
	#29	"liver stiffness":ti,ab,kw OR "hepatic stiffness":ti,ab,kw OR "LSM":ti,ab,kw
	#30	"transient elastography":ti,ab,kw OR "transient elastography technique":ti,ab,kw
	#31	"elastograph":ti,ab,kw OR "hepatic ultrasound elastography system":ti,ab,kw OR "hepatic ultrasound elastography system applicator":ti,ab,kw OR "ultrasound elastography system":ti,ab,kw OR "ultrasound elastography system applicator":ti,ab,kw OR "shear wave elastography":ti,ab,kw OR "SWE":ti,ab,kw OR "magnetic resonance elastography":ti,ab,kw OR "MRE":ti,ab,kw OR "fatty liver index":ti,ab,kw OR "FLI":ti,ab,kw OR "fatty liver score":ti,ab,kw OR "FLS":ti,ab,kw OR "fibrosis stage":ti,ab,kw OR "histological fibrosis":ti,ab,kw OR (histolog*:ti,ab,kw AND fibros*:ti,ab,kw) OR (biops*:ti,ab,kw AND fibros*:ti,ab,kw)
	#32	#14 OR #15 OR #16 OR #17 OR #18 OR #19 OR #20 OR #21 OR #22 OR #23 OR #24 OR #25 OR #26 OR #27 OR #28 OR #29 OR #30 OR #31
	#33	#8 AND #13 AND #32

Table S4. GRADE assessment of the certainty of evidence for change in ALT

Certainty assessment							№ of patients		Effect	Certainty	Importance
**№ of studies**	**Study design**	**Risk of bias**	**Inconsistency**	**Indirectness**	**Imprecision**	**Other considerations**	**Silymarin**	**Placebo**	**Absolute (95% CI)**		
**Alanine aminotransferase change (ALT change; assessed in IU/L)**											
6	randomized trials	not serious	serious	not serious	not serious	none	288	206	MD -7.25 (-11.60 lower to -2.89 lower)	⊕⊕⊕◯ Moderate	IMPORTANT

Abbreviations: CI, confidence interval; GRADE, Grading of Recommendations Assessment, Development and Evaluation; MD, mean difference.

Downgraded one level for inconsistency ($I^2 = 50.4\%$; study-specific effects in opposite directions).

Table S5. GRADE assessment of the certainty of evidence for change in AST

Certainty assessment							№ of patients		Effect		
**№ of studies**	**Study design**	**Risk of bias**	**Inconsistency**	**Indirectness**	**Imprecision**	**Other considerations**	**Silymarin**	**Placebo**	**Absolute (95% CI)**	**Certainty**	**Importance**
**Aspartate aminotransferase change (AST change; assessed in IU/L)**											
4	randomized trials	not serious	serious	not serious	not serious	none	169	137	MD -5.36 (-8.45 lower to -2.27 lower)	⨁⨁⨁◯ Moderate	IMPORTANT

Abbreviations: CI, confidence interval; GRADE, Grading of Recommendations Assessment, Development and Evaluation; MD, mean difference.

Downgraded one level for inconsistency (I^2 = 36.4%).

Table S6. GRADE assessment of the certainty of evidence for change in liver stiffness measurement

Studies	Design	Risk of bias	Inconsistency	Indirectness	Imprecision	Other considerations	Participants		Effect estimate	Certainty	Importance
							Silymarin	Control			
3	randomized trials	not serious	not serious	not serious	serious	none	106	108	MD −0.60 kPa (95% CI, −1.04 to −0.16)	⊕⊕⊕◯ Moderate	important

Abbreviations: CI, confidence interval; GRADE, Grading of Recommendations Assessment, Development and Evaluation; LSM, liver stiffness measurement; MD, mean difference.

Downgraded one level for imprecision (three trials).

Table S7. Detailed characteristics of the included randomized controlled trials (n = 37)

Study	Design	No. subjects (E/C)	Male/ female (E;C)	Age, years (E/C)	BMI, kg/m² (E/C)	Disease	Intervention	Comparator	Silymarin/ silybin dose (mg/day)	Duration (weeks)	Outcomes
**Abenavoli et al., 2015(1)**	3-arm RCT	10/10	8/2; 6/4	46 (36–59)/ 56 (45–64)	29.0 (28.0–32.0)/ 32.0 (29.0–36.0)	NAFLD	Silybin complex +diet	Diet	188	24	ALT, AST, GGT, FLI
**Abenavoli et al., 2015(1)**	3-arm RCT	10/10	8/2; 7/3	46 (36–59)/ 33 (28–44)	29.0 (28.0–32.0)/ 29.0 (27.0–32.0)	NAFLD	Silybin complex +diet	No treatment	188	24	ALT, AST, GGT, FLI
**Abenavoli et al., 2017(2)**	3-arm RCT	20/20	16/4; 12/8	46 (40–57)/ 52 (40–60)	29.0 (28.0–32.0)/ 31.0 (29.0–33.0)	NAFLD	Silymarin combination+diet and exercise	Diet and exercise	240	24	ALT, AST, GGT, FLI LSM
**Abenavoli et al., 2017(2)**	3-arm RCT	20/10	16/4; 6/4	46 (40–57)/ 33 (28–43)	29.0 (28.0–32.0)/ 29.0 (27.0–31.0)	NAFLD	Silymarin combination+diet and exercise	No treatment	240	24	ALT, AST, GGT, FLI LSM
**Ahmed et al., 2022(3)**	OL-RCT	15/15	NR	NR	NR	CHC	Silymarin+ antiviral therapy	Antiviral therapy	400	8	ALT, AST, GGT
**Aller et al., 2015(4)**	Pilot RCT	18/18	22/14‡	47.4 (11.2)‡	36.8 (7.9)/ 35.0 (7.4)	NAFLD	S.marianum extract+diet and exercise	Diet and exercise	540.3†	12	ALT, AST, GGT FLI, NFS
**Anushiravani et al., 2019(5)**	DB-PC RCT	30/30	NR	NR	25.7 (3.3)/ 26.1 (3.1)	NAFLD	Silymarin*	Placebo*	140	12	ALT, AST
**Ataee et al., 2021(6)**	TB-PC RCT	30/30	19/5; 12/5	41 (34–49)/ 40 (33–48)	28.5 (5.0)/ 29.1 (3.7)	NAFLD	Silymarin combination +diet	Placebo+diet	70	12	ALT, AST, LSM
**Cerletti et al., 2020(7)**	MC DB-RCT	55/58	35/20; 39/19	55 (12.2)/ 53 (11.3)	30.1 (3.6)/ 31.3 (5.2)	NAFLD	Silymarin combination	Control capsules	150	13	ALT, AST, GGT
**Chakrabarty et al., 2021(8)**	DB Active RCT	88‡	NR	39.3 (9.4)/ 40.6 (10.6)	29.1 (1.0)/ 29.3 (2.1)	NAFLD	Silymarin combination*	UDCA*	420	26	ALT, LSM
**Chan et al., 2017(9)**	DB-PC RCT	49/50	24/25; 22/28	49.6 (12.7)/ 50.1 (10.2)	30.0 (4.0)/ 31.0 (4.6)	NASH	Silymarin*	Placebo*	2,100	48	ALT, AST, GGT APRI, FIB-4, NFS, Histological fibrosis, LSM
**Chiurazzi et al., 2022 (10)**	RCT	36/32	16/20; 16/16	56.1 (9.9)/ 59.8 (10.9)	36.0 (8.6)/ 33.9 (6.6)	NAFLD	Silymarin combination+diet	Diet	≥200	12	ALT, AST
**Colletta et al., 2020(11)**	OL Active RCT	108/104	157/56‡	61.4 (0.4)‡	26.9 (0.1)/ 26.7 (0.1)	NAFLD	Silymarin+diet	Diet	420	26	ALT, AST, FIB-4, NFS, LSM
**Cano Contreras et al., 2025 (12)**	DB-PC RCT	25/25	5/20; 8/17	54.7 (10.5)/ 54.3 (11.5)	32.8 (6.9)/ 33.3 (4.7)	MASLD	Silymarin combination+diet	Placebo+diet	600†	24	ALT, AST, GGT, LSM

**Federico et al., 2019 (13)**	NT-RCT	60/30	29/31; 19/11	51.0 (6.0)/ 47.0 (10.0)	28.9 (6.7)/ 29.4 (4.7)	NAFLD	Silybin complex	No treatment	606†	26	ALT, AST, GGT, FIB-4, NFS, LSM
**Fried et al., 2012(14)**	MC DB-PC RCT	50/52	34/16; 41/11	54 (52–57)/ 56 (52–60)	28.5 (26.0–32.4)/29.1 (25.6–32.7)	CHC	Silymarin	Placebo	1,260	24	ALT
**Fried et al., 2012 (14)**	MC DB-PC RCT	52/52	35/17; 41/11	54 (48–58)/ 56 (51–59)	30.2 (28.1–32.9)/ 29.1 (25.6–32.7)	CHC	Silymarin	Placebo	2,100	24	ALT
**Gabbay et al., 2007 (15)**	Phase II DB-PC RCT	25/25	30/20‡	56.1‡	NR	CHC	Silymarin combination+ intravenous antioxidant therapy	Placebo+ intravenous saline	750	24	ALT, AST
**Gabbay et al., 2007 (15)**	Phase II DB-PC RCT	25/25	34/16‡	57.4‡	NR	CHC	Silymarin combination	Placebo	750	24	ALT, AST
**Gordon et al., 2006 (16)**	Cross-over RCT	24§	15/9‡	43.0 (7.0)‡	27.0 (5.0)‡	CHC	S.marianum extract	Placebo	960	12	ALT, GGT
**Gordon et al., 2006 (16)**	Cross-over RCT	24§	15/9‡	43.0 (7.0)‡	27.0 (5.0)‡	CHC	S.marianum extract	Placebo	480	12	ALT, GGT
**Hajaghamohammadi et al., 2008 (17)**	PC-RCT	25/25	16/9; 16/9	40.3/39.9	30.0/29.1	NAFLD	Silymarin	Placebo	140	8	ALT, AST
**Hashemi et al., 2009 (18)**	PC-RCT	50/50	28/22; 29/21	39.3 (11.1)/ 39.0 (10.7)	26.8 (2.7)/ 27.8 (3.8)	NAFLD/ NASH	Silymarin	Placebo	280	24	ALT, AST
**Jaffar et al., 2024 (19)**	4-arm RCT	17/17	7/9; 8/8	NR	27.1 (0.9)/ 27.4 (1.3)	NAFLD	Silymarin	Placebo	200	12.9	ALT, AST
**Jaffar et al., 2024 (19)**	4-arm RCT	18/17	8/8; 8/8	NR	27.3 (1.4)/ 27.4 (1.3)	NAFLD	Silymarin	Placebo	400	12.9	ALT, AST
**Jaffar et al., 2024 (19)**	4-arm RCT	18/17	6/10; 8/8	NR	28.1 (1.4)/ 27.4 (1.3)	NAFLD	Silymarin	Placebo	300	12.9	ALT, AST
**Jin et al., 2024 (20)**	DB-PC RCT	42/41	26/16; 23/18	45.4 (10.7)/ 40.4 (10.2)	27.0 (4.1)/ 27.6 (4.1)	MASLD	Silymarin	Placebo	103.2	24	ALT, AST, GGT FIB-4, APRI, LSM
**Kaab Omeir et al., 2025 (21)**	4-arm RCT	10/10	0/10; 0/10	43.3 (4.7)/ 40.1 (9.0)	NR	NAFLD	Silymarin	No treatment	140	8	ALT, AST
**Kaab Omeir et al., 2025 (21)**	4-arm RCT	10/10	0/10; 0/10	39.8 (7.7)/ 42.2 (8.7)	NR	NAFLD	Silymarin+exercise	Exercise	140	8	ALT, AST
**Li et al., 2024 (22)**	TB-PC RCT	60/61	30/30; 31/30	56.2 (10.0)/ 58.1 (9.6)	27.6 (2.9)/ 26.6 (2.7)	NAFLD	Silybin combination	Placebo	138	24	ALT, AST, GGT FIB-4, APRI, FLI, NFS

**Lirussi et al., 1989 (23)**	Active RCT	9/9	NR	NR	NR	Chronic active hepatitis	Silymarin	UDCA	420	24	ALT, AST, GGT
**Loguercio et al., 2012 (24)**	Phase III MC DB-PC RCT	91/88	5/9; 7/9	46.9 (10.2)/ 50.9 (9.5)	27.6 (3.6)/ 27.8 (3.4)	NAFLD	Silybin complex*	Placebo*	188	52	ALT, AST, GGT
**Loguercio et al., 2012 (24)**	Phase III MC DB-PC RCT	91/88	47/8; 43/10	40.8 (10.3)/ 44.2 (9.5)	29.9 (4.6)/ 29.3 (4.4)	NAFLD	Silybin complex*	Placebo*	188	52	ALT, AST, GGT Histological fibrosis
**Mai et al., 2025 (25)**	DB-PC 3-arm RCT	32/31	71/23‡	NR	NR	NAFLD	Silymarin combination*	Placebo*	163.2	12	ALT, AST, GGT
**Mai et al., 2025 (25)**	DB-PC 3-arm RCT	31/31	71/23‡	NR	NR	NAFLD	Silymarin*	Placebo*	163.2	12	ALT, AST, GGT
**Malaguarnera et al., 2015 (26)**	DB-PC RCT	32/32	18/14; 19/13	46.4 (6.9)/ 45.2 (6.7)	26.4 (3.7)/ 26.2 (3.8)	CHC	Silybin complex+ antiviral therapy	Placebo+ antiviral therapy	94	52	ALT, AST, Histological fibrosis
**Masoodi et al., 2013 (27)**	DB-PC RCT	50/50	31/19; 31/19	48.4 (6.8)/ 48.3 (5.5)	29.0 (3.7)/ 29.2 (3.3)	NASH	Silymarin*	Placebo*	280	12	ALT, AST
**Memon et al., 2015 (28)**	DB-PC RCT	33/31	21/12; 21/10	49.0 (9.7)/ 48.0 (8.9)	29.9 (5.8)/ 28.7 (6.8)	NAFLD	Silymarin*	Placebo*	280	12	ALT, AST
**Mirhashemi et al., 2022 (29)**	DB-PC RCT	30/30	NR	37.8 (9.9)/ 38.1 (10.0)	47.2 (7.0)/ 48.2 (7.0)	NAFLD	Silymarin*	Placebo*	560	8	ALT, AST, FIB-4, NFS, Histological fibrosis
**Navarro et al., 2019 (30)**	Phase II MC DB-PC RCT	26/25	13/13; 14/11	47.3 (10.8)/ 49.5 (10.9)	35.3 (4.8)/ 33.4 (4.8)	NASH	Silymarin	Placebo	1,260	48–50	ALT, AST, Histological fibrosis
**Navarro et al., 2019 (30)**	Phase II MC DB-PC RCT	27/25	18/9; 14/11	48.2 (11.4)/ 49.5 (10.9)	33.5 (4.3)/ 33.4 (4.8)	NASH	Silymarin	Placebo	2,100	48–50	ALT, AST, Histological fibrosis
**Rajendra et al., 2022 (31)**	DB-PC 3-arm RCT	30/30	14/16; 16/14	40.2 (10.1)/ 36.6 (7.5)	27.1 (1.6)/ 26.5 (1.5)	NAFLD	Silymarin	Placebo	320	12	ALT, AST, GGT, FLI
**Shaker et al., 2025 (32)**	Phase II MC DB-PC RCT	49/48	15/29; 13/30	40.5 (9.6)/ 40.0 (9.0)	34.1 (4.6)/ 33.9 (3.6)	Hepatic steatosis	S.marianum combination	Placebo	600†	36	ALT, AST, FIB-4, LSM
**Shaker et al., 2025 (32)**	Phase II MC DB-PC RCT	49/48	16/25; 13/30	40.5 (9.7)/ 40.0 (9.0)	34.4 (4.1)/ 33.9 (3.6)	Hepatic steatosis	S.marianum combination	Placebo	300†	36	ALT, AST, FIB-4, LSM
**Solhi et al., 2014 (33)**	PC-RCT	40/40	NR	43.6 (8.3)/ 39.4 (10.5)	27.4 (1.7)/ 27.5 (1.9)	NASH	Silymarin*	Placebo*	210	8	ALT, AST
**Taghvaei et al., 2013 (34)**	DB-PC RCT	21/20	14/7; 13/7	42.9 (11.4)/ 40.4 (12.5)	NR	NAFLD/ NASH	Silymarin*	Placebo*	280	24	ALT, AST
**Tanamly et al., 2004 (35)**	DB Active RCT	88/89	NR	44.1 (21.0–78.0)‡	NR	CHC	Silymarin	Low-dose multivitamin and mineral supplement	373.5	52	ALT

**Torres et al., 2004 (36)**	Pilot RCT	17/17	9/8; 8/9	49.0/51.0	NR	CHC	S.marianum extract	No treatment	480†	4	ALT, AST
**Zojaji et al., 2022 (37)**	DB-RCT	40/20	15/15; 10/6	49.0 (11.0)/ 44.0 (8.0)	NR	NAFLD	S.marianum combination+diet and exercise	Diet and exercise	480†	8	ALT, AST

Abbreviations: ALT, alanine aminotransferase; APRI, aspartate aminotransferase-to-platelet ratio index; AST, aspartate aminotransferase; BMI, body mass index; C, control group; CHC, chronic hepatitis C; DB, double-blind; E, experimental group; FIB-4, Fibrosis-4 index; FLI, fatty liver index; GGT, gamma-glutamyl transferase; IQR, interquartile range; LSM, liver stiffness measurement; MASLD, metabolic dysfunction-associated steatotic liver disease; MC, multicenter; NAFLD, nonalcoholic fatty liver disease; NASH, nonalcoholic steatohepatitis; NFS, NAFLD fibrosis score; NR, not reported; NT, no-treatment-controlled; OL, open-label; PC, placebo-controlled; RCT, randomized controlled trial; TB, triple-blind; SD, standard deviation; UDCA, ursodeoxycholic acid.

Data are presented as mean (SD), median (IQR), mean (range), or number of participants, as reported in the original studies. Values separated by a slash represent the experimental and control groups, respectively. Studies with multiple eligible intervention arms are presented as separate comparisons. LSM was performed using transient elastography (FibroScan®) where reported.

*Both groups received concomitant lifestyle modification (diet and/or exercise).
†The exact silymarin or silybin content was not reported; therefore, the reported doses refer to the amount of *Silybum marianum* material or preparation.
‡Data were reported for the overall study population only.
§Total number of participants randomized in the crossover trial.

Table S8. Detailed characteristics of the included non-randomized comparative studies (n = 10)

Study	Design	No. subjects (E/C)	Male/female (E;C)	Age, years (E/C)	BMI, kg/m² (E/C)	Disease	Intervention	Comparator	Silymarin /silybin dose (mg/day)	Duration (months)	Outcomes
**Akhondi-Meybodi et al., 2020 (38)**	Quasi-experimental, non-randomized	40/40	27/13; 25/15	43.7 (9.8)/ 44.2 (11.0)	29.3 (6.8)/ 30.8 (5.0)	NAFLD	Silymarin+diet	Vitamin E +diet	280	4	ALT
**Amjad et al., 2024 (39)**	Non-randomized trial	73/68	47/26; 50/18	50.26 (8.73)/ 53.25 (7.64)	27.9 (3.0)/ 26.9 (2.8)	Chronic liver disease	Silymarin+ standard care	Standard care	400	3	ALT, AST, LSM
**Curcio et al., 2020 (40)**	Controlled observational	41/40	25/16; 25/15	56.9 (14.2)/ 67.6 (11.3)	NR	NAFLD	Silymarin combination+lifestyle modification	Lifestyle modification	280	3	ALT, AST, GGT
**Falasca et al., 2008 (41)**	Observational controlled study	30/10	12/18; 4/6	56.8 (12.7)/ 59.7 (9.0)	21.8 (2.6)/ 21.6 (1.6)	CHC	Silybin complex+lifestyle modification	Lifestyle modification	188	3	ALT, AST, GGT
**Federico et al., 2017 (42)**	Prospective	17/10	14/13†	42 (11)†	29.3 (5.0)/ 29.4 (4.2)	NASH	Silybin complex	Placebo	NR	12	ALT, AST, GGT
**Freedman et al., 2011 (43)**	Prospective	170/701	133/37; 485/216	50 (47–53)/ 50 (46–54)‡	28.5 (25.8–31.8)/ 29.3 (26.3–32.8)‡	CHC	Silymarin	No silymarin use	NR	35	Histological fibrosis
**Hasan et al., 2024 (44)**	Quasi-experimental	30/30	NR	NR	NR	NAFLD	Silymarin+ standard care	Standard care	1,500	6	ALT, AST
**Rewisha et al., 2021 (45)**	Prospective controlled	150/150	88/62; 101/49	48.3 (10.8)/ 50.7 (11.2)	NR	Chronic liver disease	Silymarin	Antiviral therapy	420	9	ALT, AST, FIB-4
**Shaikh et al., 2021 (46)**	Case-control	100/100	100/0; 100/0	48.5 (7.3)/ 49.7 (10.5)	29.4 (3.6)/ 29.2 (3.9)	NAFLD	Silymarin*	Placebo*	200	3	ALT, AST
**Sorrentino et al., 2015 (47)**	Controlled observational	43/35	18/25; 24/11	56.63 (12.79)/ 55.40 (13.63)	31.8 (4.8)/ 29.7 (3.7)	NAFLD	Silybin combination+ diet and exercise	Diet and exercise	250	3	ALT, AST, GGT

Abbreviations: ALT, alanine aminotransferase; AST, aspartate aminotransferase; BMI, body mass index; C, control group; CHC, chronic hepatitis C; E, experimental group; FIB-4, Fibrosis-4 index; GGT, gamma-glutamyl transferase; IQR, interquartile range; LSM, liver stiffness measurement; NAFLD,

nonalcoholic fatty liver disease; NASH, nonalcoholic steatohepatitis; NR, not reported; SD, standard deviation.

Data are presented as mean (SD), median (IQR), mean (range), or number of participants, as reported in the original studies. Values separated by a slash represent the experimental and control groups, respectively, except in the Male/female column, where the two groups are separated by a semicolon. Combination indicates a silymarin- or silybin-containing formulation administered with one or more additional active compounds. LSM was measured using transient elastography (FibroScan®) where reported.

[*]Both groups received concomitant lifestyle modification (diet and/or exercise).
[†]Data were reported for the overall study population only.
[‡]Data are presented as median (IQR).

Table S9. Outcomes assessed in the included studies

Study	Fibrosis					Steatosis	Biochemistry			Cardiometabolic			Safety
	LSM	FIB-4	APRI	NFS	Histology	FLI	ALT	AST	GGT	Anthropometric	Glycemic	Lipids	Safety
Abenavoli et al., 2015						✓	✓	✓	✓	✓s	✓	✓	
Abenavoli et al., 2017	✓					✓	✓	✓	✓	✓	✓	✓	✓
Ahmed et al., 2022							✓	✓	✓				
Aller et al., 2015				✓		✓	✓	✓	✓	✓	✓	✓	
Anushiravani et al., 2019							✓	✓		✓	✓	✓	✓
Ataee et al., 2021	✓						✓	✓					
Cerletti et al., 2020							✓	✓	✓	✓	✓	✓	✓
Chakrabarty et al., 2021	✓						✓			✓	✓	✓	✓
Chan et al., 2017	✓	✓	✓	✓	✓		✓	✓	✓	✓	✓	✓	✓
Chiurazzi et al., 2022							✓	✓		✓	✓	✓	
Colletta et al., 2020	✓	✓		✓			✓	✓			✓		✓
Contreras et al., 2025	✓						✓	✓	✓	✓	✓	✓	✓
Federico et al., 2019	✓	✓		✓			✓	✓	✓	✓	✓	✓	
Fried et al., 2012							✓						✓
Gabbay et al., 2007							✓	✓					✓
Gordon et al., 2006							✓		✓				✓
Hajaghamohammadi et al., 2008							✓	✓		✓			
Hashemi et al., 2009							✓	✓					✓
Jaffar et al., 2024							✓	✓		✓		✓	✓
Jin et al., 2024	✓	✓	✓				✓	✓	✓	✓	✓	✓	✓
Kaab Omeir et al., 2025							✓	✓					
Li et al., 2024		✓	✓	✓		✓	✓	✓	✓	✓	✓	✓	
Lirussi et al., 1989							✓	✓	✓				
Loguercio et al., 2012					✓		✓	✓	✓	✓	✓		✓

Mai et al., 2025						✓	✓	✓			✓	
Malaguarnera et al., 2015				✓		✓	✓					
Masoodi et al., 2013						✓	✓		✓			
Memon et al., 2015						✓	✓			✓	✓	
Mirhashemi et al., 2022		✓	✓	✓		✓	✓		✓			✓
Navarro et al., 2019				✓		✓	✓			✓		✓
Rajendra et al., 2022					✓	✓	✓	✓		✓	✓	✓
Shaker et al., 2025	✓	✓				✓	✓				✓	✓
Solhi et al., 2014						✓	✓					
Taghvaei et al., 2013						✓	✓					
Tanamly et al., 2004						✓						✓
Torres et al., 2004						✓	✓					✓
Zojaji et al., 2022						✓	✓			✓	✓	
Akhondi-Meybodi et al., 2020						✓						
Amjad et al., 2024	✓					✓	✓					
Curcio et al., 2020						✓	✓	✓			✓	✓
Falasca et al., 2008						✓	✓	✓	✓	✓	✓	✓
Federico et al., 2017						✓	✓	✓	✓	✓		
Freedman et al., 2011				✓								
Hasan et al., 2024						✓	✓		✓	✓	✓	
Rewisha et al., 2021		✓				✓	✓					
Shaikh et al., 2021						✓	✓					
Sorrentino et al., 2015						✓	✓	✓	✓	✓	✓	✓

Abbreviations: ALT, alanine aminotransferase; APRI, aspartate aminotransferase-to-platelet ratio index; AST, aspartate aminotransferase; CAP, controlled attenuation parameter; FIB-4, Fibrosis-4 index; FLI, Fatty Liver Index; GGT, gamma-glutamyl transferase; LSM, liver stiffness measurement; NFS, NAFLD fibrosis score.

Anthropometric outcomes included body weight, BMI, waist circumference, or related measures. Glycemic outcomes included fasting glucose, HbA1c, insulin, or insulin-resistance indices. Lipid outcomes included total cholesterol, triglycerides, LDL cholesterol, or HDL cholesterol. Safety included adverse events, serious adverse events, or treatment discontinuation.

A check mark indicates that the corresponding outcome was assessed or reported in the original study. The table indicates outcome availability and does not denote the direction or statistical significance of the treatment effect.

Table S10. Study characteristics and liver stiffness measurement findings in trials with treatment durations of ≥24 weeks

Study	Design	N	Disease	Dose	Formulation	Comparator	Duration	LSM
Abenavoli et al. (2017)	3-arm RCT	20/20	NAFLD	240	Combination	Diet and exercise	24	LSM decreased in both groups, with no statistically significant between-group difference.
Abenavoli et al. (2017)	3-arm RCT	20/10	NAFLD	240	Combination	None	24	LSM decreased in the diet-plus-antioxidant group and increased in the no-treatment group, with a statistically significant between-group difference.
Chakrabarty et al. (2021)	DB Active RCT	88[b]	NAFLD	420	Combination	UDCA	26	LSM decreased in the silymarin–choline group; the direction of the between-group comparison was not clearly reported.
Chan et al. (2017)	DB-PC RCT	49/50	NASH	2,100	Single	Placebo	48	LSM decreased more in the silybin group than in the placebo group, but the between-group difference in mean LSM change was not statistically significant.
Colletta et al. (2020)	OL Active RCT	108/104	NAFLD	420	Single	Diet	26	LSM decreased in both groups, with no statistically significant additional reduction in the silymarin-plus-diet group compared with the diet-only group.
Cano Contreras et al. (2025)	DB-PC RCT	25/25	MASLD	600	Combination	Placebo + diet	24	LSM decreased in both groups; the between-group comparison was not clearly reported.
Federico et al. (2019)	NT-RCT	60/30	NAFLD	606	Combination	None	26	LSM decreased after treatment; the direction of the between-group comparison was not clearly reported.
Jin et al. (2024)	DB-PC RCT	42/41	MASLD	103.2	Single	Placebo	24	LSM decreased in the silymarin group and increased in the placebo group, with a statistically significant between-group difference.
Shaker et al. (2025)	Phase II MC DB-PC RCT	49/48	Hepatic steatosis	600	Combination	Placebo	36	LSM decreased from baseline in the high-dose group, but the between-group difference was not statistically significant.
Shaker et al. (2025)	Phase II MC DB-PC RCT	49/48	Hepatic steatosis	300	Combination	Placebo	36	LSM decreased from baseline in the low-dose group, but the between-group difference was not statistically significant.

Abbreviations: LSM, liver stiffness measurement.

LSM findings were summarized according to the reported within-group changes and between-group comparisons. Studies were not classified solely according to statistical significance.

(a) ALT

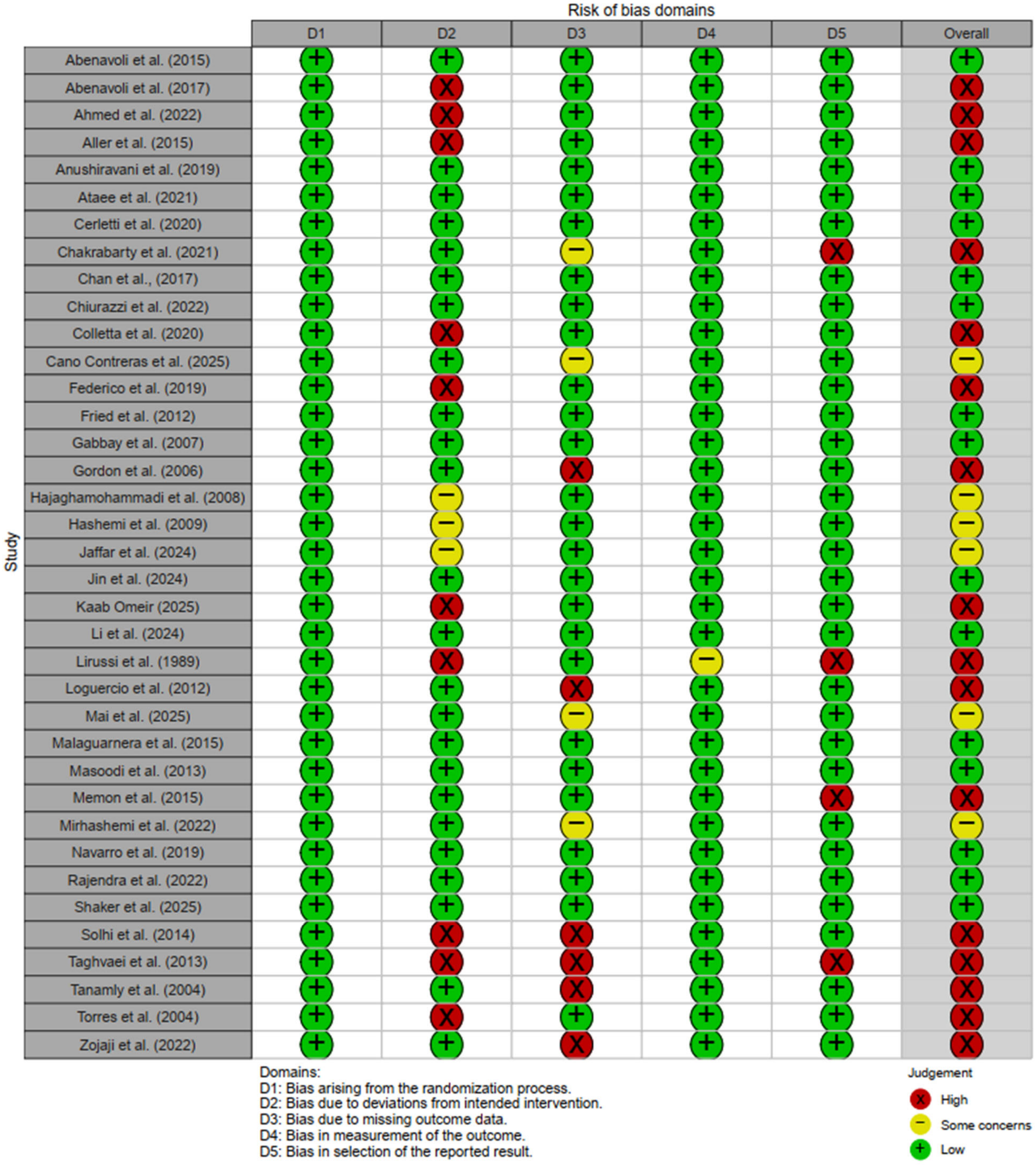
Risk of bias domains
D1
D2
D3
D4
D5
Overall
Study
Abenavoli et al. (2015)
Abenavoli et al. (2017)
Ahmed et al. (2022)
Aller et al. (2015)
Anushiravani et al. (2019)
Ataee et al. (2021)
Cerletti et al. (2020)
Chakrabarty et al. (2021)
Chan et al., (2017)
Chiurazzi et al. (2022)
Colletta et al. (2020)
Cano Contreras et al. (2025)
Federico et al. (2019)
Fried et al. (2012)
Gabbay et al. (2007)
Gordon et al. (2006)
Hajaghamohammadi et al. (2008)
Hashemi et al. (2009)
Jaffar et al. (2024)
Jin et al. (2024)
Kaab Omeir (2025)
Li et al. (2024)
Lirussi et al. (1989)
Loguercio et al. (2012)
Mai et al. (2025)
Malaguarnera et al. (2015)
Masoodi et al. (2013)
Memon et al. (2015)
Mirhashemi et al. (2022)
Navarro et al. (2019)
Rajendra et al. (2022)
Shaker et al. (2025)
Solhi et al. (2014)
Taghvaei et al. (2013)
Tanamly et al. (2004)
Torres et al. (2004)
Zojaji et al. (2022)
Domains:
D1: Bias arising from the randomization process.
D2: Bias due to deviations from intended intervention.
D3: Bias due to missing outcome data.
D4: Bias in measurement of the outcome.
D5: Bias in selection of the reported result.
Judgement
High
Some concerns
Low

(b) AST

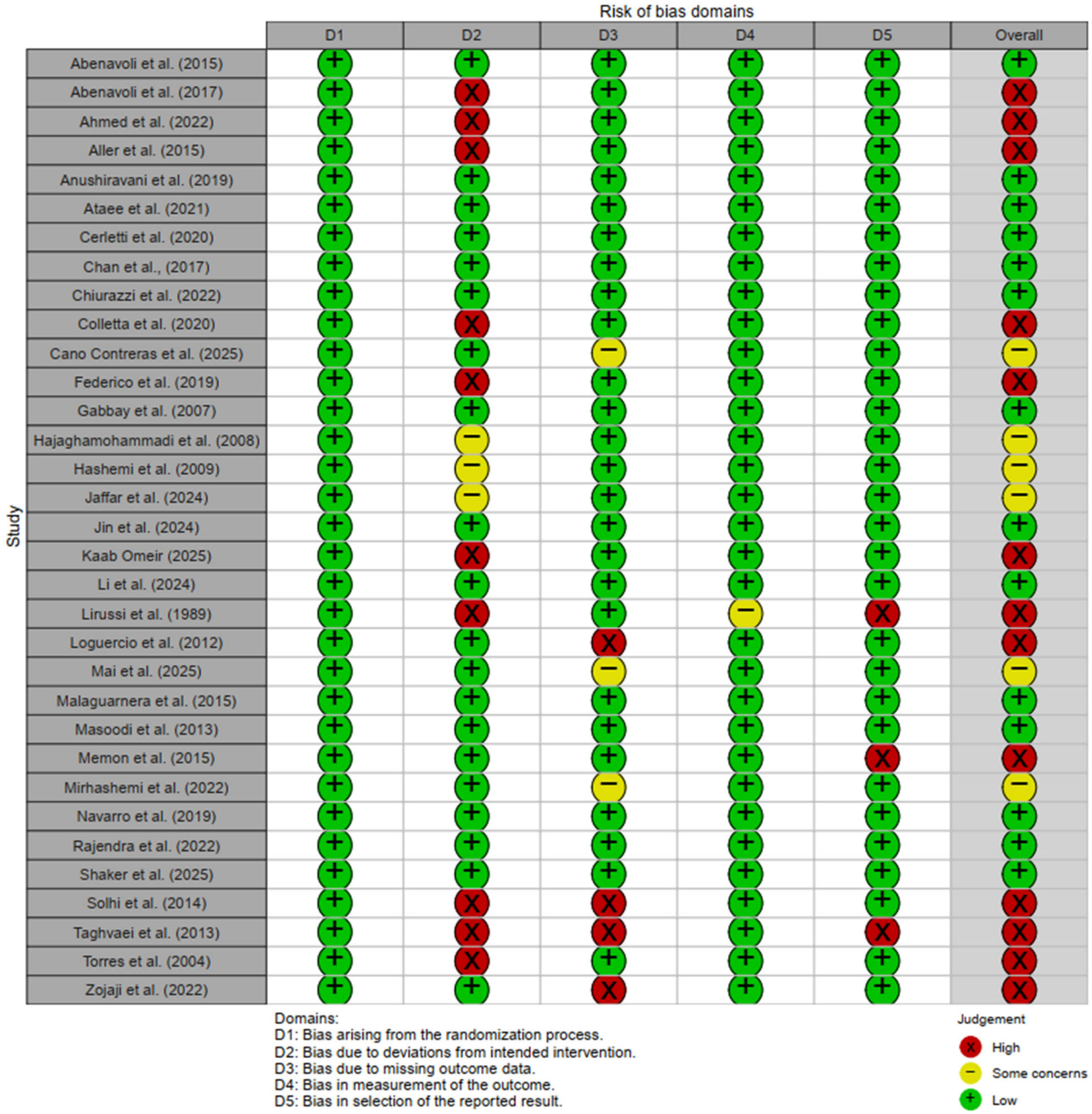
Risk of bias domains
D1
D2
D3
D4
D5
Overall
Study
Abenavoli et al. (2015)
Abenavoli et al. (2017)
Ahmed et al. (2022)
Aller et al. (2015)
Anushiravani et al. (2019)
Ataee et al. (2021)
Cerletti et al. (2020)
Chan et al., (2017)
Chiurazzi et al. (2022)
Colletta et al. (2020)
Cano Contreras et al. (2025)
Federico et al. (2019)
Gabbay et al. (2007)
Hajaghamohammadi et al. (2008)
Hashemi et al. (2009)
Jaffar et al. (2024)
Jin et al. (2024)
Kaab Omeir (2025)
Li et al. (2024)
Lirussi et al. (1989)
Loguercio et al. (2012)
Mai et al. (2025)
Malaguarnera et al. (2015)
Masoodi et al. (2013)
Memon et al. (2015)
Mirhashemi et al. (2022)
Navarro et al. (2019)
Rajendra et al. (2022)
Shaker et al. (2025)
Solhi et al. (2014)
Taghvaei et al. (2013)
Torres et al. (2004)
Zojaji et al. (2022)
Domains:
D1: Bias arising from the randomization process.
D2: Bias due to deviations from intended intervention.
D3: Bias due to missing outcome data.
D4: Bias in measurement of the outcome.
D5: Bias in selection of the reported result.
Judgement
High
Some concerns
Low

(c) GGT

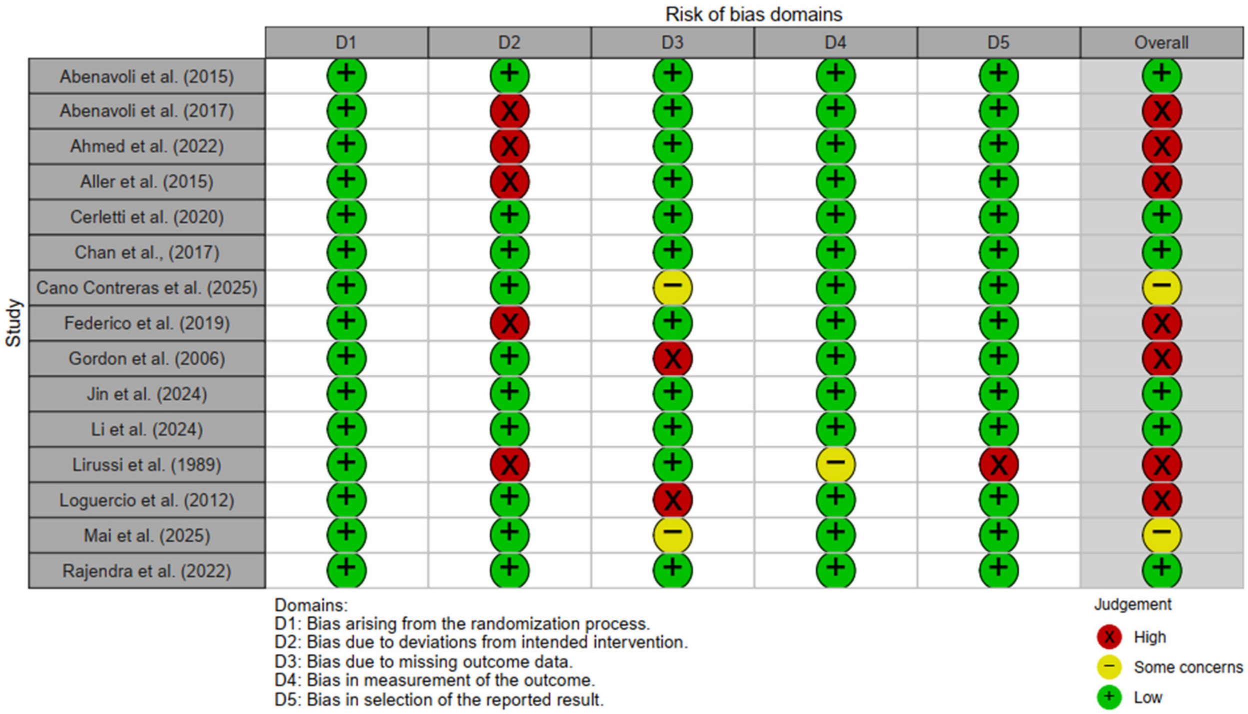
Risk of bias domains
D1
D2
D3
D4
D5
Overall
Study
Abenavoli et al. (2015)
Abenavoli et al. (2017)
Ahmed et al. (2022)
Aller et al. (2015)
Cerletti et al. (2020)
Chan et al., (2017)
Cano Contreras et al. (2025)
Federico et al. (2019)
Gordon et al. (2006)
Jin et al. (2024)
Li et al. (2024)
Lirussi et al. (1989)
Loguercio et al. (2012)
Mai et al. (2025)
Rajendra et al. (2022)
Domains:
D1: Bias arising from the randomization process.
D2: Bias due to deviations from intended intervention.
D3: Bias due to missing outcome data.
D4: Bias in measurement of the outcome.
D5: Bias in selection of the reported result.
Judgement
High
Some concerns
Low

(d) FIB−4

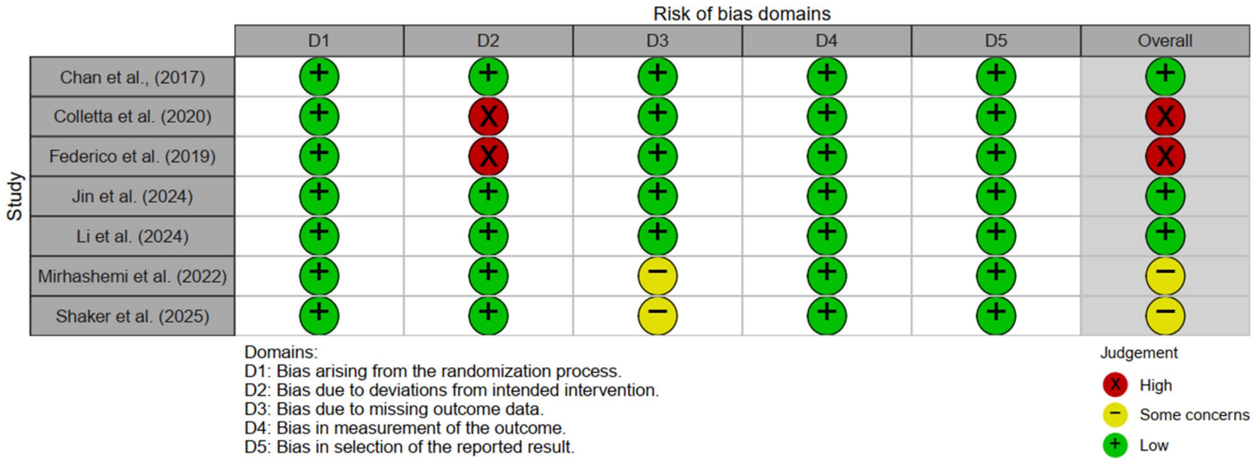
Risk of bias domains
D1
D2
D3
D4
D5
Overall
Study
Chan et al., (2017)
Colletta et al. (2020)
Federico et al. (2019)
Jin et al. (2024)
Li et al. (2024)
Mirhashemi et al. (2022)
Shaker et al. (2025)
Domains:
D1: Bias arising from the randomization process.
D2: Bias due to deviations from intended intervention.
D3: Bias due to missing outcome data.
D4: Bias in measurement of the outcome.
D5: Bias in selection of the reported result.
Judgement
High
Some concerns
Low

(e) LSM

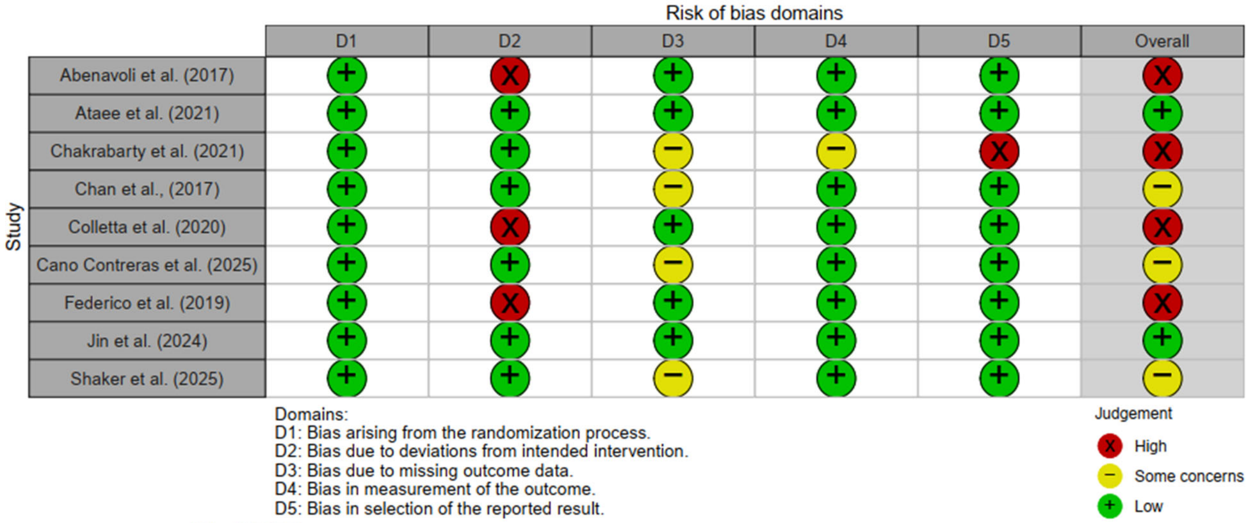

(f) APRI

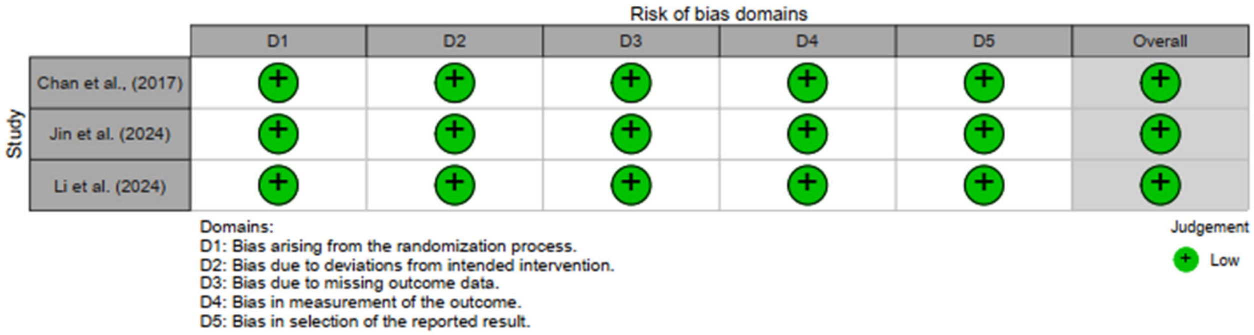

(g) NFS

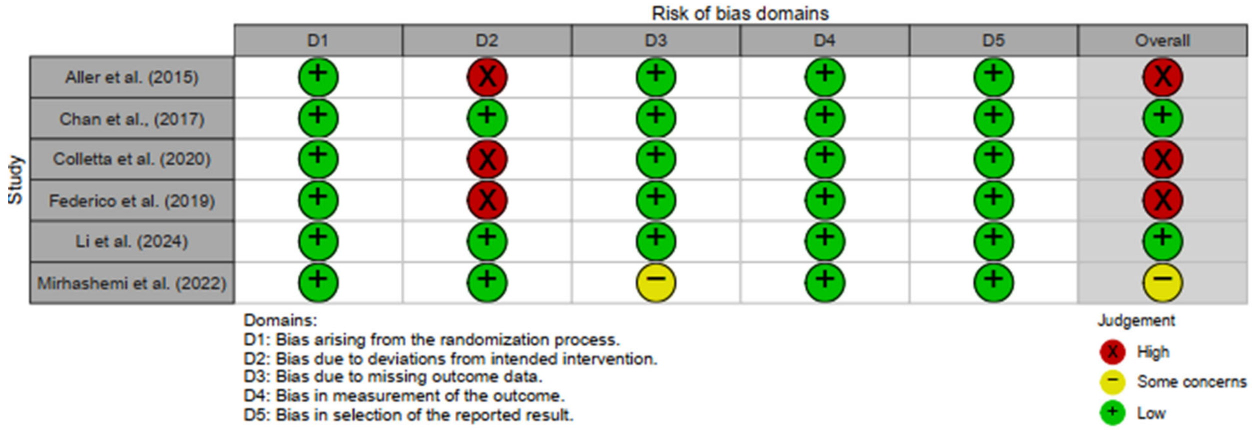

(h) Histological fibrosis

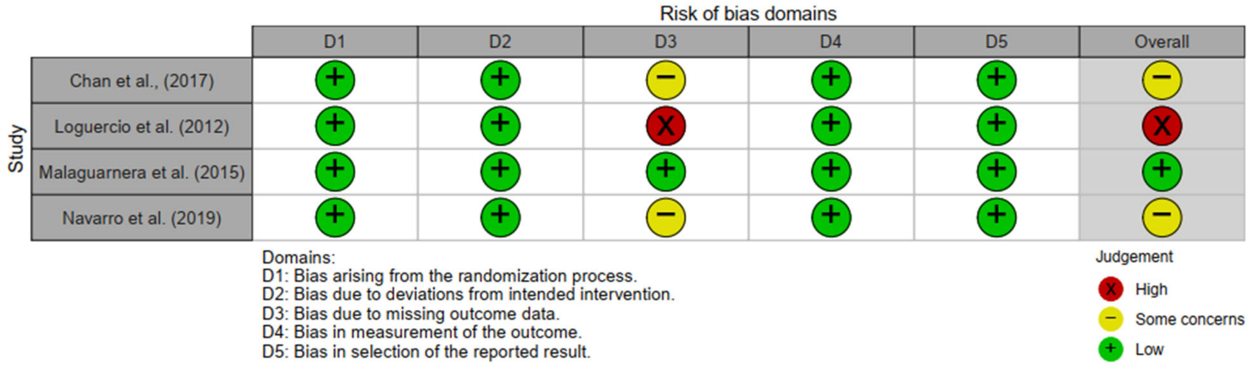

(i) FLI

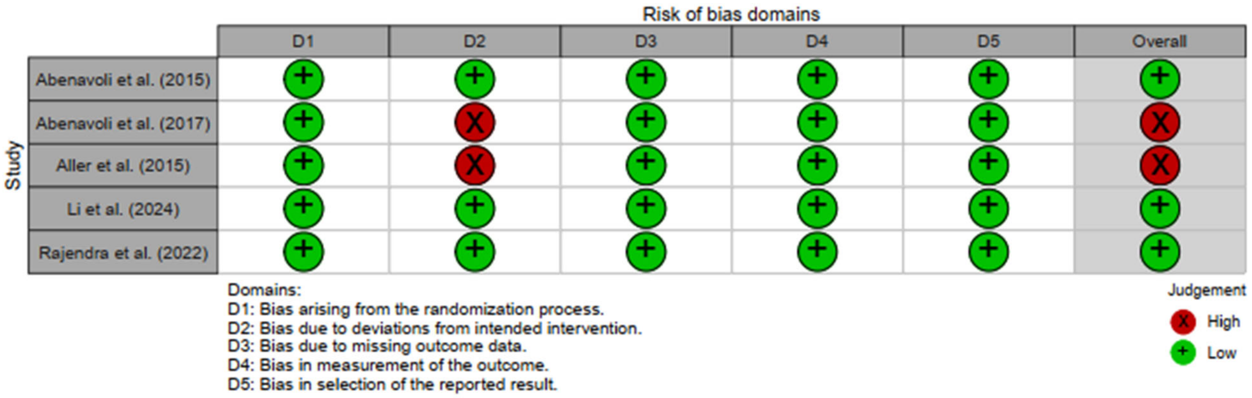

Supplementary Figure S1. Outcome-specific risk-of-bias assessment of randomized controlled trials using the Cochrane Risk of Bias 2 tool. Assessments are presented separately for (a) ALT, (b) AST, (c) GGT, (d) FIB-4, (e) LSM, (f) APRI, (g) NFS, (h) histological fibrosis, and (i) FLI.

Green indicates low risk, yellow indicates some concerns, and red indicates high risk of bias.

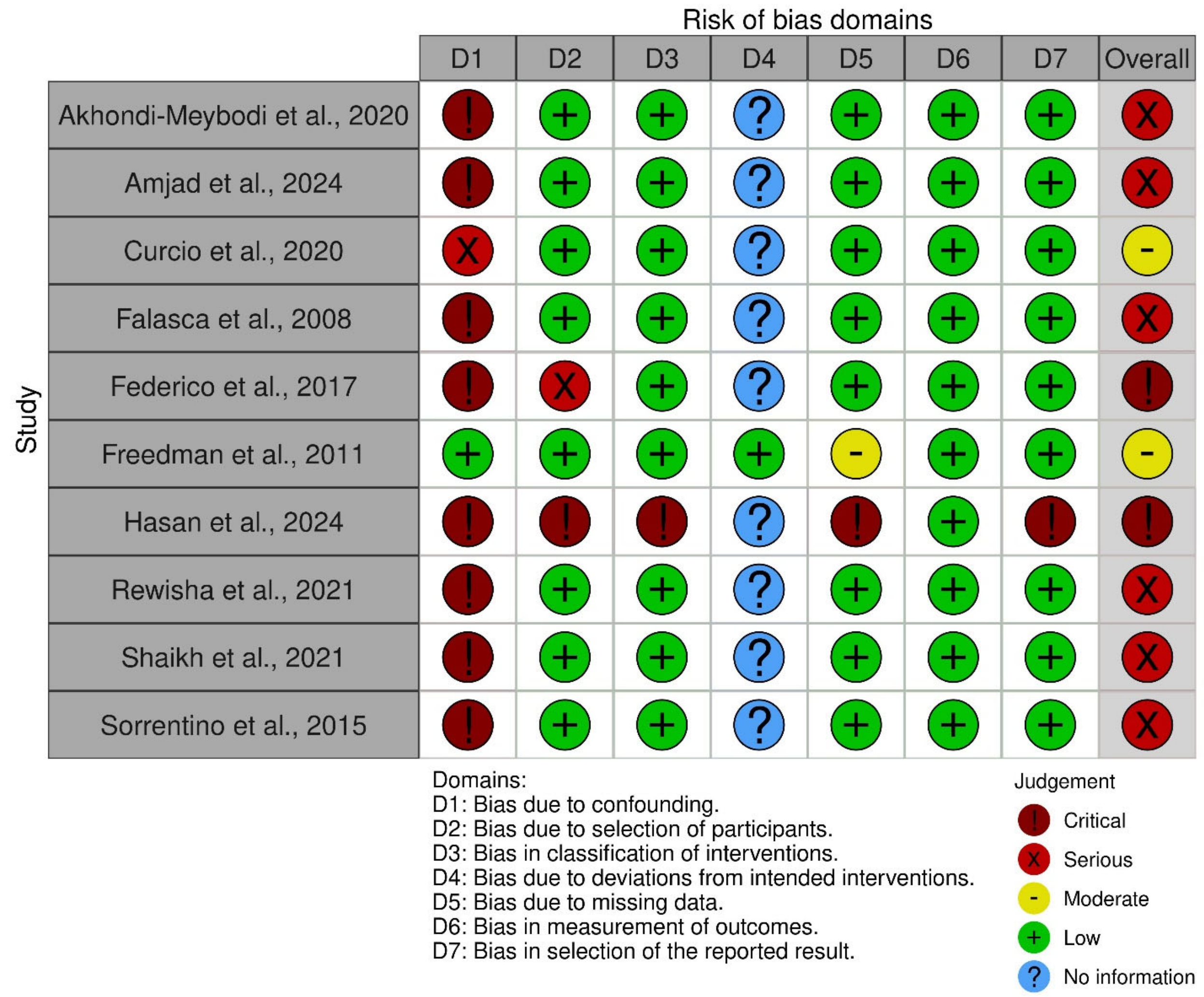

Supplementary Figure S2. Risk-of-bias assessment of the included non-randomized studies using the ROBINS-I tool.

Judgments were categorized as low, moderate, serious, critical, or no information, according to the ROBINS-I framework.

ROBINS-I assessments were conducted at the study–outcome level; however, no judgements changed because the available risk-of-bias information was consistent across outcomes within each study.

REFERENCES

1. Abenavoli L, Greco M, Nazionale I, Peta V, Milic N, Accattato F, et al. Effects of Mediterranean diet supplemented with silybin-vitamin E-phospholipid complex in overweight patients with non-alcoholic fatty liver disease. Expert Rev Gastroenterol Hepatol. 2015;9(4):519–27.
2. Abenavoli L, Greco M, Milic N, Accattato F, Foti D, Gulletta E, et al. Effect of Mediterranean Diet and Antioxidant Formulation in Non-Alcoholic Fatty Liver Disease: A Randomized Study. Nutrients. 2017;9(8).
3. Ahmed S, Ullah N, Parveen S, Javed I, Jalil NAC, Murtey MD, et al. Effect of Silymarin as an Adjunct Therapy in Combination with Sofosbuvir and Ribavirin in Hepatitis C Patients: A Miniature Clinical Trial. Oxid Med Cell Longev. 2022;2022:9199190.
4. Aller R, Izaola O, Gómez S, Tafur C, González G, Berroa E, et al. Effect of silymarin plus vitamin E in patients with non-alcoholic fatty liver disease. A randomized clinical pilot study. Eur Rev Med Pharmacol Sci. 2015;19(16):3118–24.
5. Anushiravani A, Haddadi N, Pourfarmanbar M, Mohammadkarimi V. Treatment options for nonalcoholic fatty liver disease: a double-blinded randomized placebo-controlled trial. Eur J Gastroenterol Hepatol. 2019;31(5):613–7.
6. Ataee Z, Vahabzadeh M, Mazloumi KSS, Rahimi HR, MOSANNEN MH, Ziaee M. Triple therapy with garlic, silymarin and curcumin in non-alcoholic fatty liver disease: a randomized, placebo-controlled clinical trial. 2021.
7. Cerletti C, Colucci M, Storto M, Semeraro F, Ammollo CT, Incampo F, et al. Randomised trial of chronic supplementation with a nutraceutical mixture in subjects with non-alcoholic fatty liver disease. Br J Nutr. 2020;123(2):190–7.

8. Chakrabarty P, Ghosh S, Mukherjee M, Adhikary R, Mukherjee S, Majumder S, et al. Silymarin-choline combination versus ursodeoxycholic acid in non-alcoholic fatty liver disease: A randomised double-blind clinical trial. Gut. 2021;70(SUPPL 3):A52.

9. Chan WK NMN, Mahadeva S. A Randomized Trial of Silymarin for the Treatment of Nonalcoholic Steatohepatitis. Clin Gastroenterol Hepatol. 2017;15(12):1940–9.e8.

10. Chiurazzi M, Cacciapuoti N, Di Lauro M, Nasti G, Ceparano M, Salomone E, et al. The Synergic Effect of a Nutraceutical Supplementation Associated to a Mediterranean Hypocaloric Diet in a Population of Overweight/Obese Adults with NAFLD. Nutrients. 2022;14(22).

11. Colletta C, Colletta A, Placentino G. Lifestyle and silymarin: a fight against liver damage in NAFLD associated - prediabetic disease. J Diabetes Metab Disord. 2020;19(2):883–94.

12. Cano Contreras AD, Del Rocío Francisco M, Vargas Basurto JL, Gonzalez-Gomez KD, Amieva-Balmori M, Roesch Dietlen F, et al. Effect of alpha-lipoic acid and Silybum marianum supplementation with a Mediterranean diet on metabolic dysfunction-associated steatosis. World J Hepatol. 2025;17(1):101704.

13. Federico A, Dallio M, Masarone M, Gravina AG, Di Sarno R, Tuccillo C, et al. Evaluation of the Effect Derived from Silybin with Vitamin D and Vitamin E Administration on Clinical, Metabolic, Endothelial Dysfunction, Oxidative Stress Parameters, and Serological Worsening Markers in Nonalcoholic Fatty Liver Disease Patients. Oxid Med Cell Longev. 2019;2019:8742075.

14. Fried MW, Navarro VJ, Afdhal N, Belle SH, Wahed AS, Hawke RL, et al. Effect of silymarin (milk thistle) on liver disease in patients with chronic hepatitis C unsuccessfully treated with interferon therapy: a randomized controlled trial. Jama. 2012;308(3):274–82.

15. Gabbay E, Zigmond E, Pappo O, Hemed N, Rowe M, Zabrecky G, et al. Antioxidant

therapy for chronic hepatitis C after failure of interferon: Results of phase II randomized, double-blind placebo controlled clinical trial. World Journal of Gastroenterology. 2007;13(40):5317–23.

16. Gordon A, Hobbs DA, Bowden DS, Bailey MJ, Mitchell J, Francis AJ, et al. Effects of Silybum marianum on serum hepatitis C virus RNA, alanine aminotransferase levels and well-being in patients with chronic hepatitis C. J Gastroenterol Hepatol. 2006;21(1 Pt 2):275–80.

17. Hajaghamohammadi AA, Ziaee A, Rafiei R. The Efficacy of Silymarin in Decreasing Transaminase Activities in Non-Alcoholic Fatty Liver Disease: A Randomized Controlled Clinical Trial. Hepatitis Monthly. 2008;8(3):191–5.

18. Hashemi SJ, Hajiani E, Sardabi EH. A Placebo-Controlled Trial of Silymarin in Patients with Nonalcoholic Fatty Liver Disease. Hepatitis Monthly. 2009;9(4):265–70.

19. Jaffar HM, Bader Ul Ain H, Tufail T, Hanif A, Malik T. Impact of silymarin-supplemented cookies on liver enzyme and inflammatory markers in non-alcoholic fatty liver disease patients. Food Sci Nutr. 2024;12(10):7273–86.

20. Jin Y, Wang X, Chen K, Chen Y, Zhou L, Zeng Y, et al. Silymarin decreases liver stiffness associated with gut microbiota in patients with metabolic dysfunction-associated steatotic liver disease: a randomized, double-blind, placebo-controlled trial. Lipids Health Dis. 2024;23(1):239.

21. Kaab Omeir S HA, Jassemi Zergani F, Alizadeh AA. Effect of Eight weeks of Pilates training with Silymarin consumption on liver enzyme levels in women with non-alcoholic fatty liver disease. Metabolism and Exercise. **2025**, 15, 68–87.

22. Li BY, Xi Y, Liu YP, Wang D, Wang C, Chen CG, et al. Effects of Silybum marianum, Pueraria lobate, combined with Salvia miltiorrhiza tablets on non-alcoholic fatty liver disease in adults: A triple-blind, randomized, placebo-controlled clinical trial. Clinical Nutrition ESPEN. 2024;63:2–12.

23. Lirussi F, Orlando R, Iemmolo RM, Zappala F, Nassuato G, Okolicsanyi L. Effect of UDCA and silymarin on liver function in patients with chronic active hepatitis with cirrhosis: a prospective study. Journal of hepatology. 1989;9(Suppl 1):S184.

24. Loguercio C, Andreone P, Brisc C, Brisc MC, Bugianesi E, Chiaramonte M, et al. Silybin combined with phosphatidylcholine and vitamin E in patients with nonalcoholic fatty liver disease: a randomized controlled trial. Free Radic Biol Med. 2012;52(9):1658–65.

25. Mai M, Wang Y, Wang D, Ruan Y, Mo B, Li Z, et al. Effects of Silymarin Combined with Herbal Medicine on Th17/Treg Cell Imbalance and Inflammatory Response in Patients with Nonalcoholic Fatty Liver Disease: A Randomized, Double-Blind, Placebo-Controlled Clinical Trial. Hepatitis Monthly. 2025;25(1).

26. Malaguarnera M, Motta M, Vacante M, Malaguarnera G, Caraci F, Nunnari G, et al. Silybin-vitamin E-phospholipids complex reduces liver fibrosis in patients with chronic hepatitis C treated with pegylated interferon α and ribavirin. Am J Transl Res. 2015;7(11):2510–8.

27. Masoodi M, Rezadoost A, Panahian M, Vojdanian M. Effects of Silymarin on Reducing Liver Aminotransferases in Patients with Nonalcoholic Fatty Liver Diseases. Iranian journal of gastroenterology & hepatology (GOVARESH). 2013;18(3):181–5.

28. Memon IA, Akbar M, Bhurgri AN. Effect of silymarin therapy on liver aminotransferase in non-alcoholic fatty liver disease. Medical Forum Monthly. 2015;26(12):46–9.

29. Mirhashemi SH, Hakakzadeh A, Yeganeh FE, Oshidari B, Rezaee SP. Effect of 8 Weeks milk thistle powder (silymarin extract) supplementation on fatty liver disease in patients candidates for bariatric surgery. Metabol Open. 2022;14:100190.

30. Navarro VJ, Belle SH, D'Amato M, Adfhal N, Brunt EM, Fried MW, et al. Silymarin in non-cirrhotics with non-alcoholic steatohepatitis: A randomized, double-blind, placebo controlled

trial. PLoS ONE. 2019;14(9).

31. Pothula Rajendra VK, Kurapati S, Balineni SK, Gogineni NT. A blend of Sphaeranthus indicus flower head and Terminalia chebula fruit extracts reduces fatty liver and improves liver function in non-alcoholic, overweight adults. Functional Foods in Health and Disease. 2022;12(7):361–79.

32. Shaker MK, Hassany M, Eysa B, Adel A, Zidan A, Mohamed S. The activity of a herbal medicinal product of Phyllanthus niruri and Silybum marianum powdered extracts (Heptex®) in patients with apparent risk factors for nonalcoholic steatohepatitis: a phase II, multicentered, randomized, double-blind, placebo-controlled clinical trial. BMC Complement Med Ther. 2025;25(1):8.

33. Solhi H, Ghahremani R, Kazemifar AM, Hoseini Yazdi Z. Silymarin in treatment of non-alcoholic steatohepatitis: A randomized clinical trial. Caspian J Intern Med. 2014;5(1):9–12.

34. Taghvaei T, Bahar A, Hosseini V, Maleki I, Kasrai M. Efficacy of silymarin on treatment of nonalcoholic steatohepatitis. Journal of Mazandaran University of Medical Sciences. 2013;23(98):164–71.

35. Tanamly MD, Tadros F, Labeeb S, Makld H, Shehata M, Mikhail N, et al. Randomised double-blinded trial evaluating silymarin for chronic hepatitis C in an Egyptian village: study description and 12-month results. Dig Liver Dis. 2004;36(11):752–9.

36. Torres M, Rodríguez-Serrano F, Rosario DJ, Rodríguez-Perez F, Toro DH. Does Silybum marianum play a role in the treatment of chronic hepatitis C? P R Health Sci J. 2004;23(2):69–74.

37. Zojaji SA, Mozaffari HM, Ghaderi P, Zojaji F, Hadjzadeh MAR, Seyfimoqadam M, et al. Efficacy of an herbal compound in decreasing steatosis and transaminase activities in non-alcoholic fatty liver disease: A randomized clinical trial. Brazilian Journal of Pharmaceutical

Sciences. 2022;58.

38. Akhondi-Meybodi M BM, Namazi A. The efficacy of silymarin and vitamin E in non-alcoholic fatty liver disease: a clinical trial. 2020.

39. Amjad MZ, Hassan MU, Rehman M, Shamim A, Zeeshan HM, Iftikhar Z, et al. Impact of Silymarin Supplementation on Liver Function and Enzyme Profiles in Diverse Chronic Liver Disease Etiologies. Cureus. 2024;16(12):e76313.

40. Curcio A, Romano A, Cuozzo S, Nicola AD, Grassi O, Schiaroli D, et al. Silymarin in Combination with Vitamin C, Vitamin E, Coenzyme Q10 and Selenomethionine to Improve Liver Enzymes and Blood Lipid Profile in NAFLD Patients. Medicina (Kaunas). 2020;56(10).

41. Falasca K, Ucciferri C, Mancino P, Vitacolonna E, De Tullio D, Pizzigallo E, et al. Treatment with silybin-vitamin E-phospholipid complex in patients with hepatitis C infection. J Med Virol. 2008;80(11):1900–6.

42. Federico A, Conti V, Russomanno G, Dallio M, Masarone M, Stiuso P, et al. A Long-term Treatment with Silybin in Patients with Non-alcoholic Steatohepatitis Stimulates Catalase Activity in Human Endothelial Cells. In Vivo. 2017;31(4):609–18.

43. Freedman ND, Curto TM, Morishima C, Seeff LB, Goodman ZD, Wright EC, et al. Silymarin use and liver disease progression in the Hepatitis C Antiviral Long-Term Treatment against Cirrhosis trial. Aliment Pharmacol Ther. 2011;33(1):127–37.

44. Hasan MK. Effect of Silymarin on Hepatic Steatosis and Stiffness in patient with Non-Alcoholic Fatty Liver Disease (NAFLD). 2024.

45. Rewisha E, Salman T, Alhaddad O, Raia GA, Naguib M, Rashad S, et al. Hyaluronic acid as a potential marker for assessment of fibrosis regression after direct acting antiviral drugs in chronic hepatitis C patients. Clin Exp Hepatol. 2021;7(3):320–7.

46. Shaikh KR, Shaikh S, Ata MA, Memon A, Soomro UA, Siddiqui SS. Therapeutic efficacy of silymarin on liver aminotransferases in patients with nonalcoholic fatty liver disease. Rawal Medical Journal. 2021;46(4):761–4.

47. Sorrentino G, Crispino P, Coppola D, De Stefano G. Efficacy of Lifestyle Changes in Subjects with Non-Alcoholic Liver Steatosis and Metabolic Syndrome May Be Improved with an Antioxidant Nutraceutical: A Controlled Clinical Study. Drugs in R and D. 2015;15(1):21–5.
